# The Hsp70-Chaperone Machines in Bacteria

**DOI:** 10.3389/fmolb.2021.694012

**Published:** 2021-06-07

**Authors:** Matthias P. Mayer

**Affiliations:** Center for Molecular Biology of Heidelberg University (ZMBH), DKFZ-ZMBH-Alliance, Heidelberg, Germany

**Keywords:** molecular chaperone, Hsp70, HscA, HscC, allostery, protein folding, stress response

## Abstract

The ATP-dependent Hsp70s are evolutionary conserved molecular chaperones that constitute central hubs of the cellular protein quality surveillance network. None of the other main chaperone families (Tig, GroELS, HtpG, IbpA/B, ClpB) have been assigned with a comparable range of functions. Through a multitude of functions Hsp70s are involved in many cellular control circuits for maintaining protein homeostasis and have been recognized as key factors for cell survival. Three mechanistic properties of Hsp70s are the basis for their high versatility. First, Hsp70s bind to short degenerate sequence motifs within their client proteins. Second, Hsp70 chaperones switch in a nucleotide-controlled manner between a state of low affinity for client proteins and a state of high affinity for clients. Third, Hsp70s are targeted to their clients by a large number of cochaperones of the J-domain protein (JDP) family and the lifetime of the Hsp70-client complex is regulated by nucleotide exchange factors (NEF). In this review I will discuss advances in the understanding of the molecular mechanism of the Hsp70 chaperone machinery focusing mostly on the bacterial Hsp70 DnaK and will compare the two other prokaryotic Hsp70s HscA and HscC with DnaK.

## Introduction

**FIGURE 1:**
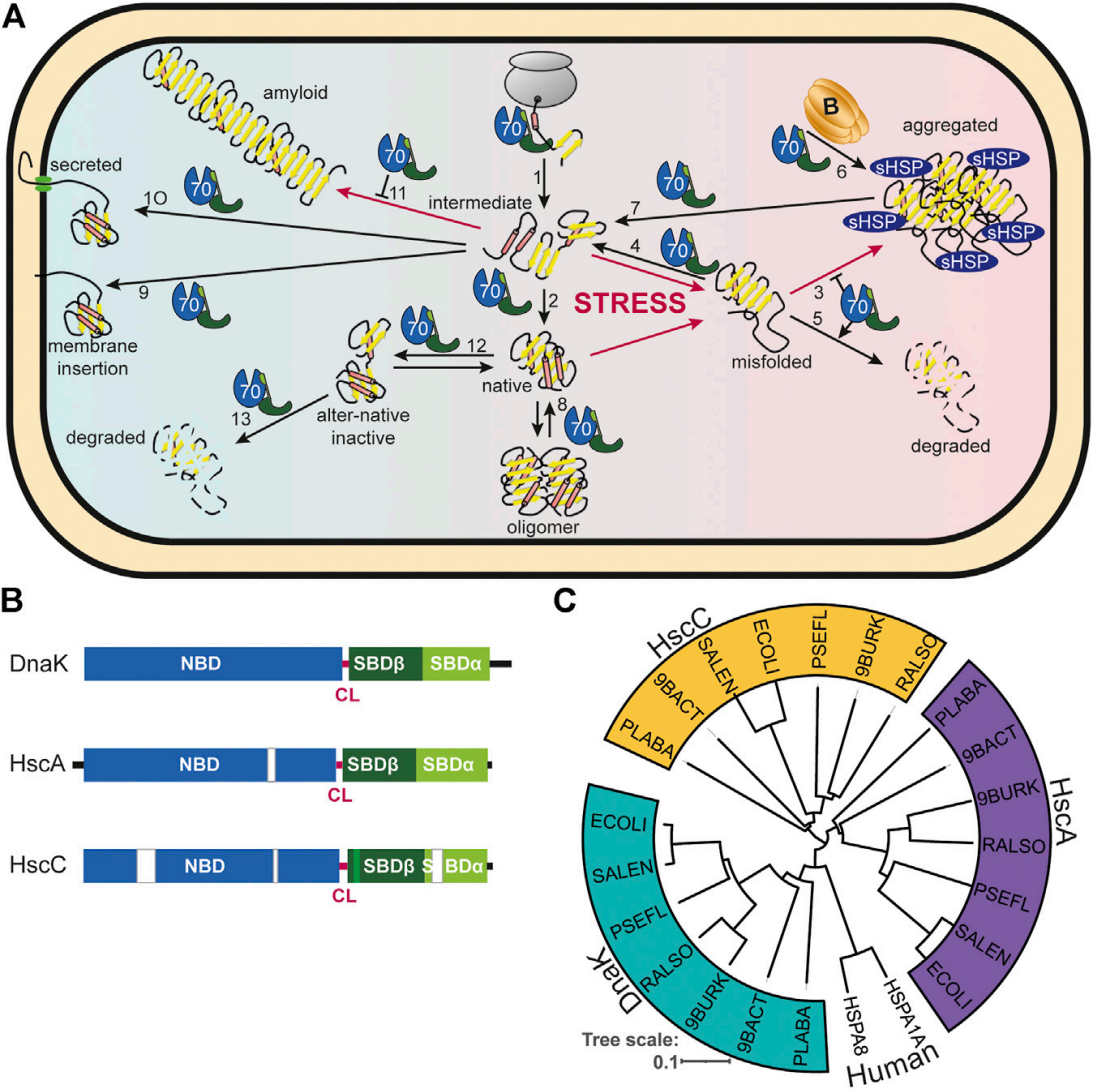
Diversity of Hsp70s and their functions in prokaryotic cells. **(A)**, Diversity of functions of Hsp70s under optimal growth conditions (middle to left) and upon exposure to environmental and physiological stress (middle to right). Hsp70/DnaK (70) assists *de-novo*-folding of proteins, interacting with nascent chains already at the ribosome (1) and with folding intermediates after release from the ribosome (2). Folding intermediates and even native proteins may misfold, in particular under stress conditions, and become aggregation prone. Hsp70 prevents aggregation (3) and refolds the misfolded protein by unfolding (4) or target it for degradation (5). Under severe stress conditions protein aggregates are formed by coaggregation with sHSPs. Hsp70 targets ClpB (B) to the aggregates (6). ClpB solubilizes the aggregated proteins that are subsequently refolded by Hsp70 (7). Hsp70 disassembles homo-and heterooligomeric protein complexes like RepE-dimers and the λO·λP·DnaB complex (8). Proteins destined for insertion into the plasma membrane (e.g., DjlC) (9), or secretion into the periplasmic space (e.g., PhoA or curli) (10) are guided by Hsp70 and prevented from forming aggregates or amyloid fibrils (curli) (11) in the cytoplasm. Hsp70 also interacts with some native proteins like the heat shock transcription factor σ^32^ to keep them in an alter-native inactive conformation (12) and target them to degradation (13). **(B)**, Domain organization of the three types of Hsp70s that exist in prokaryotes, DnaK, HscA and HscC. NBD, nucleotide binding domain (blue); CL, conserved linker (magenta); SBDβ, β-sandwich domain (dark green; light green: insertion in HscCs); SBDα, α-helical lid domain (chartreuse); black lines, C-terminal intrinsically disordered tails (for HscA also N-terminal extension); white bars, larger deletions in NBD and SBD of HscA and HscC as compared to DnaK. **(C)**, Phylogenetic tree of different prokaryotic clades that contain organisms which have all three Hsp70s. ECOLI, *Escherichia coli* (γ-Proteobacteria); SALEN, *Salmonella enteritidis* (γ-Proteobacteria); PSEFL, *Pseudomonas fluorescence* (γ-Proteobacteria); RALSO, *Ralstonia solanacaearum* (β-proteobacteria); 9BURK, *Paraburkholderia fungorum* (β-proteobacteria); 9BACT, *Acidobacteria bacterium* (unclassified Acidobacteria); PLABA, *Planctomycetes bacterium* (unclassified Planctomycetes); HSPA1A, human Hsp70; HSPA8, human Hsc70 (for comparison). A more extensive phylogenetic tree can be found in Barriot et al. (2020).

The ATP-dependent 70 kDa heat shock proteins (Hsp70s) are without doubt the most versatile of all chaperones and involved in many diverse folding processes in the cell (Meimaridou et al., 2009; Clerico et al., 2015). To name just a few of their functions in bacteria, Hsp70s assist *de-novo*-folding of proteins interacting with nascent chains already at the ribosome (Deuerling et al., 1999; Calloni et al., 2012), prevent aggregation of stress denatured proteins (Mogk et al., 1999), and solubilize protein aggregates (Goloubinoff et al., 1999) (Figure 1A). They disassemble native protein complexes like, for example, the λO-λP-DnaB complex during replication of bacteriophage λ (Zylicz et al., 1989), the homodimeric replication initiation proteins RepA of P1 phages (Wickner et al., 1991) and RepE of the mini-F plasmids (Ishiai et al., 1994), and the dimeric RctB replication initiator of chromosome 2 in *Vibrio cholerae* (Jha et al., 2017). Hsp70s are important for the insertion of tail-anchored proteins into the plasma membrane (Peschke et al., 2018). Hsp70s prevent formation of amyloids in the cytoplasm and assist secretion of the functional amyloid curli that is necessary for biofilm formation and cell adhesion (Evans et al., 2011; Sugimoto et al., 2018). Hsp70s are also involved in virulence of many pathogenic bacteria [for review see (Ghazaei, 2017)]. For example, swimming, swarming, and twitching motility, cell adherence, expression of virulence factors and their injection into host cells, engulfment of the pathogen into phagocytosomes, and survival in endosomes were shown to depend on Hsp70s (Köhler et al., 1996; Hanawa et al., 2002; Singh et al., 2007; Okuda et al., 2017; Collet et al., 2018). Most importantly, Hsp70s are involved in the regulation of the heat shock response in many proteobacteria (Matsui et al., 2008; Kobayashi et al., 2011; Schumann, 2016; Schramm et al., 2017).

This enormous versatility of Hsp70s is based in three basic principles. First, with their tweezer-like polypeptide substrate binding domain (SBD) Hsp70s bind short degenerative sequence motifs found in most proteins with high frequency. Thus, the actions of Hsp70s are not limited by size or conformation of their clients, as long as the sequence motif is accessible. Second, binding of Hsp70s to client proteins is regulated by an intricate allosteric mechanism through ATP binding and hydrolysis in their nucleotide binding domain (NBD). Third, Hsp70s are targeted to client proteins by cochaperones of the J-domain protein (JDP) family, for example DnaJ, the prototype JDP, and for generalist Hsp70s the lifetime of the Hsp70-client complex is regulated by the nucleotide exchange factor (NEF) GrpE. In addition, Hsp70 cooperate with other families of chaperones, like the small heat shock proteins (sHSPs, inclusion body binding proteins, IbpA, IbpB) (Veinger et al., 1998; Żwirowski et al., 2017), the oxidative stress activated Hsp33 (Winter et al., 2005), the chaperonin (GroEL-GroES) (Langer et al., 1992), the Hsp90 (Genest et al., 2011; Morán Luengo et al., 2018), and the Hsp100/ClpB (Goloubinoff et al., 1999) chaperones and take over clients from them or relay clients to them.

Despite their involvement in such a large number of protein-folding processes, Hsp70s are not strictly essential in many bacteria and two free-living bacterial species of the Aquificales order, *Desulfobacterium thermolithotrophum* and *Thermovibrio ammonificans*, have been described that do not encode for any Hsp70, nor any of its JDP cochaperones or GrpE, and have apparently lost these genes in the course of evolution (Warnecke, 2012). These strictly anaerobic, chemolithotrophic organisms have a growth temperature optimum of 70 and 75°C, respectively, and have a significantly reduced genome size that is only about one third the size of the *Escherichia coli* genome. Apparently, proteins can evolve to fold efficiently even at high temperatures without the assistance of the Hsp70 chaperone system. Consistently, the Hsp70 system is also absent in hyperthermophilic archaea, whereas it is present in their mesophilic relatives. However, the absence of the Hsp70 system also comes with a price. Like Hsp90s (Rutherford and Lindquist, 1998; Queitsch et al., 2002) and Hsp60s (Maisnier-Patin et al., 2005), Hsp70s buffer the accumulation of mutations in the genome and therefore increase the evolvability of the organism (Aguilar-Rodríguez et al., 2016; Kadibalban et al., 2016). In fact, proteins that depend strongly on Hsp70, as defined by Calloni and colleagues (Calloni et al., 2012), evolve faster than proteins that do not depend on Hsp70 for folding (Aguilar-Rodríguez et al., 2016; Kadibalban et al., 2016).

The model organism *Escherichia coli* harbors three structurally and functionally distinct Hsp70s: DnaK that is found in all prokaryotes, with the exceptions mentioned above, and that is the best-studied of all Hsp70s; HscA, an Hsp70 that is not found in many bacteria and that is specialized to assist the assembly of iron sulfur clusters (Vickery and Cupp-Vickery, 2007); and HscC, a specialized Hsp70 that confers resistance to Cd^2+^-ions and UV irradiation through an unknown mechanism (Kluck et al., 2002) (Figure 1B). The differences in sequence and structure between the three Hsp70s is quite remarkable including some deletions and insertions in otherwise highly conserved regions (Figure 1B). In fact, *E. coli* DnaK shares more sequence identity with human Hsp70 (48.4/61.9% identity/similarity), than with *E. coli* HscA (39.3/56.6%) or *E. coli* HscC (27.8/46.8%), and HscA and HscC are also only distantly related to each other (28.9/46.7%). This becomes even more apparent in a phylogenetic tree where DnaK, HscA and HscC segregate in clearly independent branches (Figure 1C) [see (Barriot et al., 2020) for a more extensive phylogenetic analysis]. This sequence divergence may have significant mechanistic distinctions but have only been investigated to a limited extent. HscA and HscC are not found outside the prokaryotic kingdom, though, in some fungi, Hsp70s that are specialized for iron sulfur cluster assembly emerged through convergent evolution (Schilke et al., 2006; Kleczewska et al., 2020).

Deletion of *dnaK* in *E. coli* leads to cold and heat sensitivity with a very restricted growth temperature range between 20 and 35°C and cells exhibit a filamentous phenotype (Paek and Walker, 1987). The *∆dnaK* strain tends to accumulate a second site suppressor mutation in the *rpoH* gene down-regulating amount or activity of the heat shock transcription factor σ^32^, indicating that unchecked σ^32^ leads to a detrimental imbalance in transcription (Bukau and Walker, 1990). Cells with the second site suppressor are still temperature sensitive but are not anymore filamentous at 30°C. Similar observations were made for the α-proteobacterium *Caulobacter crescentus* (Schramm et al., 2017). Deletion of *hscA* increased the doubling time of *E. coli* by twofold in rich medium but not in minimal medium and combined deletion of *hscA* and *dnaK* increased the doubling time threefold as compared to wild type *E. coli* (Hesterkamp and Bukau, 1998). However, plating efficiency was not altered. Deletion of *hscC* did not decrease viability of *E. coli* at 30 and 37°C in rich medium and the deletion of either *hscA* or *hscC* or both together do not aggravate the temperature sensitivity phenotype of a *∆dnaK* strain (Kluck et al., 2002). Neither *hscA* nor *hscC* could complement the temperature sensitivity phenotype of a *∆dnaK* strain when overexpressed and overexpression of either *hscA* or *dnaK* in a ∆hscC strain does not alleviate increased Cd^2+^ sensitivity, clearly showing the distinction between the different Hsp70s in *E. coli* (Kluck et al., 2002).

Since DnaK is not only physiologically more important in *E. coli*, more widespread in the prokaryotic kingdom, and more closely related to human Hsp70, it has been for many years the paradigm for Hsp70s and its molecular mechanism was investigated in great detail. In the following I will mainly focus on *E. coli* DnaK. Insights into structure and mechanism of Hsp70s gained through studies on yeast and mammalian Hsp70 are included when there is reason to believe that these features are also valid for the prokaryotic Hsp70 systems or to point out particular distinctions.

## Hsp70 Domain Structure and Functional Cycle

### Structure of DnaK-Like Hsp70s

**FIGURE 2:**
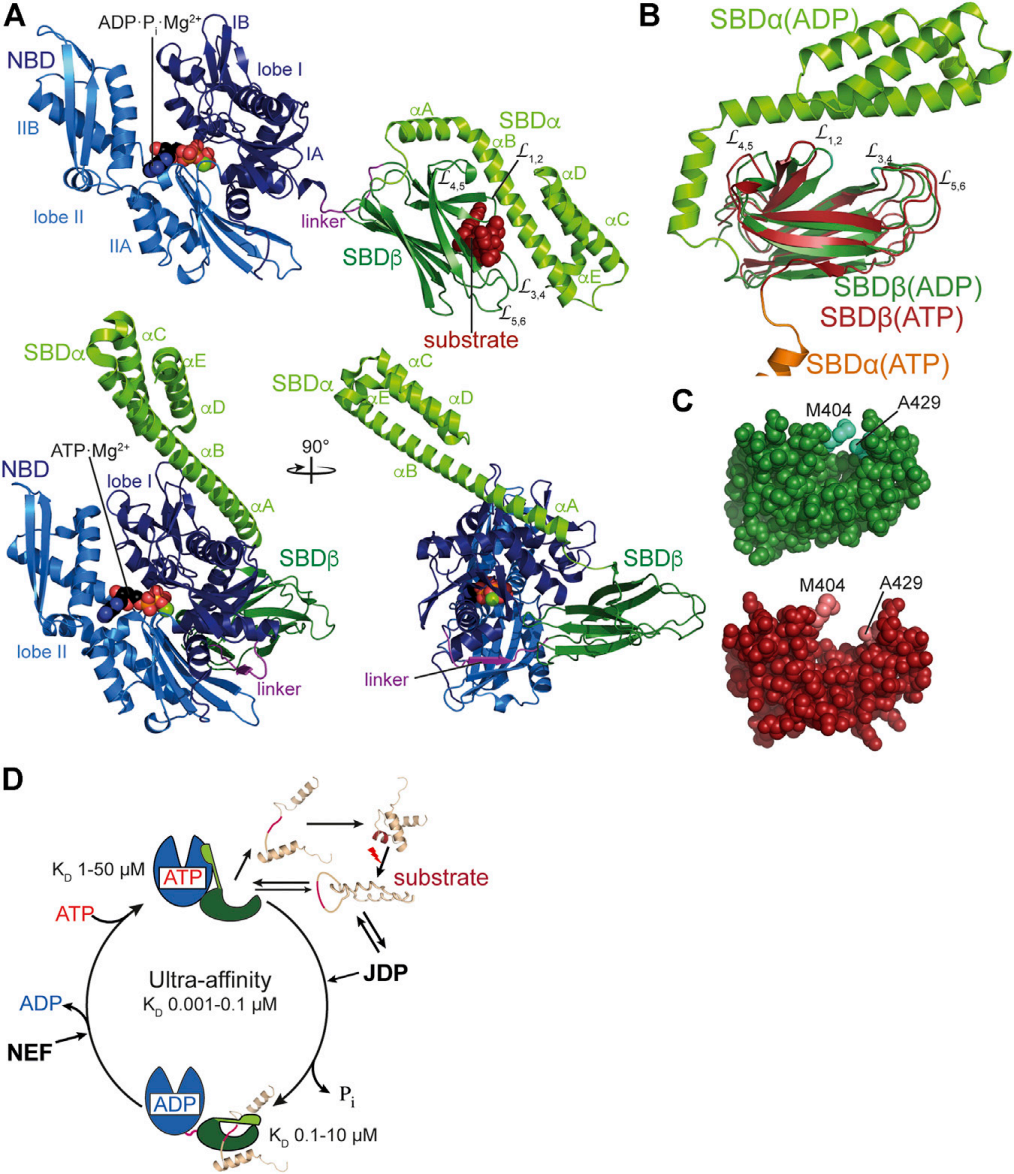
Structure and functional cycle of Hsp70s. **(A)**, Cartoon representation of DnaK in the ADP·P_i_·Mg^2+^-bound, SBD-closed and domain-undocked conformation (upper panel; PDB ID 2KHO (Bertelsen et al., 2009)) and ATP·Mg^2+^-bound, SBD-open, domain-docked conformation [lower panels in two orientations; 4B9Q (Kityk et al., 2012)]. NBD lobe I (subdomains IA and IB), dark blue; NBD lobe II (subdomains IIA and IIB), marine blue; conserved linker, magenta; SBDβ, dark green; SBDα, chartreuse; ADP and ATP in space-filling representation colored according to atoms with carbon, black, oxygen, red, nitrogen blue and phosphorus, orange, Mg^2+^, green; substrate peptide, dark red in space-filling representation. **(B)**, Overlay of the structures of the SBD of the ADP-bound, closed [SBDβ, dark green and SBDα, chartreuse; 1DKX (Zhu et al., 1996)] and the ATP-bound, open conformation [SBDβ, dark red and SBDα, orange, cut for space reasons; 4B9Q (Kityk et al., 2012)]. Substrate enclosing loops L_1,2_, L_3,4_, L_4,5_, and L_5,6_ are labeled. **(C)**, space-filling representation of the crystal structure of the SBDβ in the closed, substrate-bound conformation (upper panel, dark green), and the open conformation in the ATP-bound state (lower panel, dark red); arch forming residues M404 and A429 are indicated. **(D)**, ATPase cycle of Hsp70s. Partially folded or misfolded substrate polypeptides associate with and dissociate from Hsp70 with high rates in the ATP-bound open conformation. Substrates may also interact with the J-domain protein (JDP) co-chaperone. Substrate and JDP synergistically trigger ATP hydrolysis and transition to the closed, domain-undocked conformation. During this process substrate unfolding may occur. Alternatively or in addition, Hsp70 may select the more unfolded species from a equilibrium of different conformations. At physiological ATP concentrations nucleotide exchange is rate-limiting for substrate release. Nucleotide exchange factors (NEF) catalyze ADP release, and ATP rebinding stimulates substrate release that subsequently might fold into the native state or might rebind to Hsp70 for another folding cycle. Dark red indicate Hsp70 binding site. K_D_ values for typical high-affinity binding peptides to ADP and ATP bound states are indicated. Association of the substrate to the ATP-bound state with subsequent ATP hydrolysis creates a non-equilibrium situation called ultra-affinity (De Los Rios and Barducci, 2014).

*Bona fide* Hsp70s like DnaK consist of an N-terminal nucleotide binding domain (NBD) of 385 amino acids connected *via* a conserved linker to a polypeptide substrate binding domain (SBD) of around 240 residues (Figure 2A). The NBD is built up of four subdomains (IA, IB, IIA, IIB) arranged in two lobes that are separated by a deep cleft at the bottom of which the nucleotide binds with nanomolar affinity (Flaherty et al., 1990). ATP binding and hydrolysis involves rotation of the lobes relative to each other (Kityk et al., 2012). The SBD is subdivided in a β-sandwich subdomain (SBDβ) of around 110 residues, an α-helical subdomain (SBDα) of approximately 100 residues and a C-terminal intrinsically disordered region of some 30 residues. The polypeptide binding cleft is formed by the two twisted four-stranded β-sheets of the SBDβ and two concentric pairs of upward protruding loops (Zhu et al., 1996). In the high affinity conformation, the SBDα docks onto two faces of the SBDβ, stabilizing the inner loops (L_1,2_, L_4,5_) and forms a latch of hydrogen bonds and a salt bridge with the outer loops (L_3,4_, L_5,6_). Therefore, the SBDα acts like a lid over the substrate binding groove and restricts substrate association and dissociation (Mayer et al., 2000; Moro et al., 2004). This arrangement allows for the tweezer-like binding to short, extended polypeptide segments of around five residues with a central hydrophobic sidechain inserting into a deep hydrophobic pocket that seems to be tailored for leucine. Upon ATP binding to the NBD, the SBDα dissociates from the SBDβ and both subdomains dock onto different faces of the NBD resulting in a scissors like opening of the β-sandwich and peptide enclosing loops (Figures 2B,**C**), increasing the peptide association and dissociation rates by 100 and 1,000-fold, respectively, decreasing the affinity for peptide substrates by 10–50-fold (Schmid et al., 1994; Mayer et al., 2000; Kityk et al., 2012; Qi et al., 2013). ATP binding and hydrolysis, thus, allosterically regulate the affinity of Hsp70s for peptide and protein substrates (Figure 2D). It is important to note that, although the ability to prevent aggregation of a misfolded protein was the original definition of a molecular chaperone, Hsp70s alone are generally not particularly apt to do so: In the ADP-bound or nucleotide-free state the association rates to binding segments are too low (ca. 10^4^ M^−1^s^−1^ corresponding to a half-life for complex formation of ca. 1–2 min at 1 µM concentration and 30°C) to compete efficiently with the aggregation reaction and in the ATP bound state the affinity for binding sites is too low (1–50 µM for good binders) to reduce the free concentration of aggregation prone species enough to prevent the concentration dependent oligomerization process of misfolded client proteins. Therefore, Hsp70s need to encounter their misfolded protein clients in the ATP bound low-affinity conformation of the SBD with high substrate association rates and then hydrolyze ATP to trap the client in the ADP bound high-affinity conformation (Figure 2D). Consequently, ATP hydrolysis is essential for Hsp70 action as has been demonstrate for several Hsp70s (Wawrzynów et al., 1995; Elefant and Palter, 1999; Barthel et al., 2001; Lagaudrière-Gesbert et al., 2002; Kumar and Tiwari, 2018). However, intrinsic ATP hydrolysis rates of Hsp70s are generally very low amounting to one molecule of ATP hydrolyzed every 3–30 min (McCarty et al., 1995; Silberg and Vickery, 2000; Kluck et al., 2002). This intrinsic ATPase rate is stimulated by the client protein in synergism with a J-domain cochaperone to rates that allow binding to clients on the seconds timescale. Association of the client with the high association rates of the ATP bound state and subsequent rapid ATP hydrolysis and transition to the ADP bound state with low client dissociation rates creates a non-equilibrium situation that increases the apparent affinity by several orders of magnitude, a property that was coined ultra-affinity (De Los Rios and Barducci, 2014). Of note, in the nucleotide-free or ADP-bound state DnaK is not always in the high-affinity conformation but the SBDα lid occasionally opens allowing for association and dissociation of bound polypeptides (Mayer et al., 2000; Kityk et al., 2012; Lai et al., 2017). Conversely, in the ATP-bound state DnaK is not always in the low-affinity conformation and the SBDα may detach from the NBD occasionally. Therefore, in both nucleotide-bound states Hsp70s are in an equilibrium between different conformations with the nucleotides biasing the rates of transition.

### Allosteric Mechanism

**FIGURE 3:**
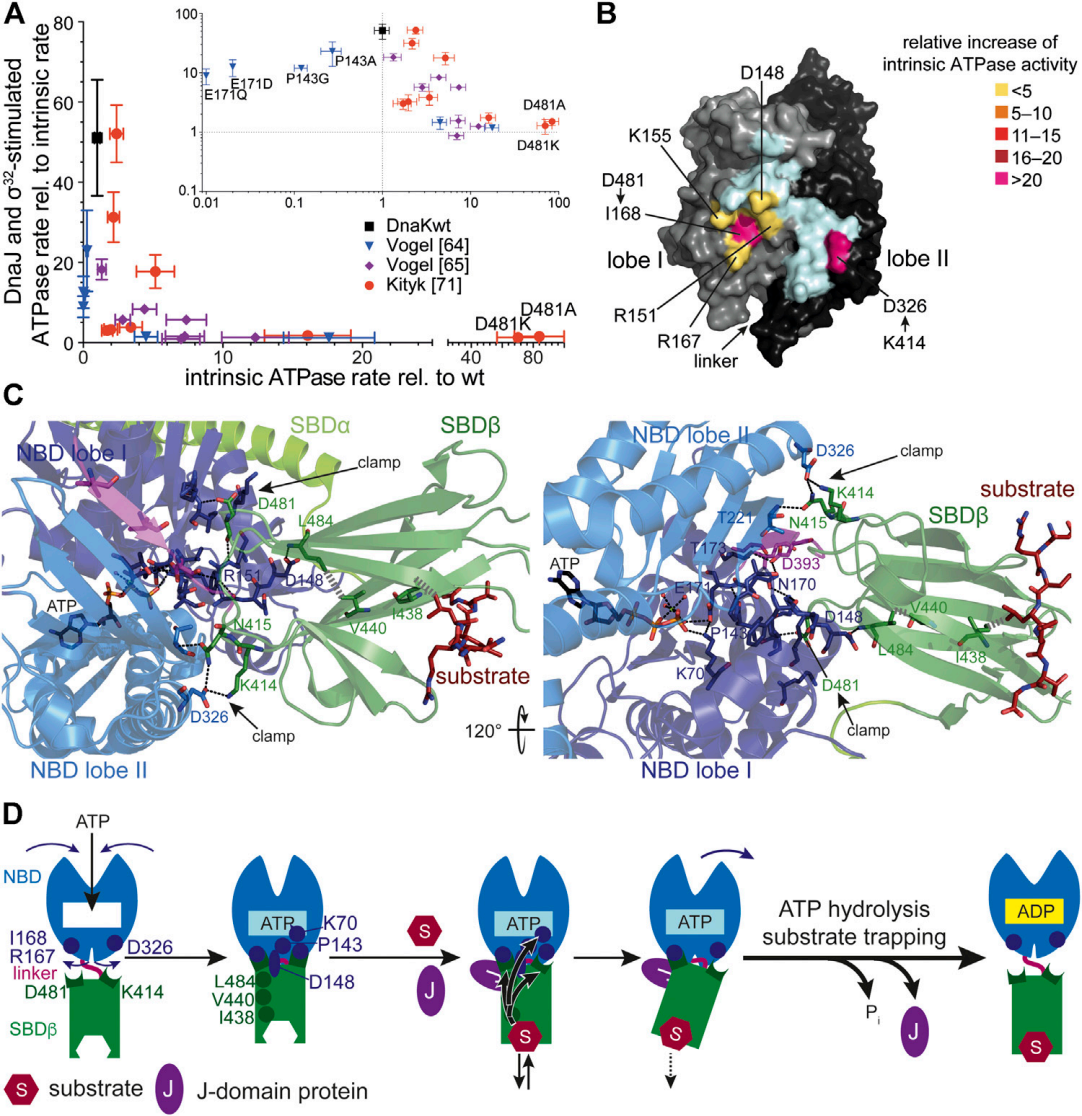
Allostery in Hsp70s. **(A)**, Amino acid replacements outside the catalytic pocket that impair interdomain communication increase the intrinsic ATPase rate. A signature for allosteric proficiency of DnaK variants is the synergistic stimulation of DnaK’s ATPase rate by DnaJ and its protein client σ^32^. Thus, single turnover ATPase rates of wild type and mutant DnaK proteins in the presence of 50 nM DnaJ and 1 µM σ^32^ is plotted vs. their intrinsic ATPase rate. Defects in allostery reduce the DnaJ-σ^32^-stimulated ATPase rate. Inset, same data on logarithmic scales. Data taken from (Vogel et al., 2006a; Vogel et al., 2006b; Kityk et al., 2015). **(B)**, Surface representation of the NBD of DnaK in the ATP-bound state (4B9Q) with lobe I and lobe II colored in gray and black, respectively, and the interface to which the SBDβ docks in light cyan, except for the indicated residues known to be involved in allostery themselves or contacted by residues of the SBDβ known to be involved in allostery. These are colored according to the relative increase of intrinsic ATPase activity when these residues are replaced themselves by alanine or if their pendant in the SBDβ is replaced by alanine (D481A) or isoleucine (K414I) [modified from (Mayer, 2018)]. **(C)**, Intramolecular pathways of allostery. Polar (black dashed lines) and non-polar (gray hatched lines) interactions from the substrate to the catalytic center for ATP hydrolysis. Indicated are contacts (D481→I168 and K414→D326, N415→T221) that fix the NBD lobes in the rotated, ATP hydrolysis-incompetent state (clamp) (Kityk et al., 2015). Right panel rotate by 120° as compared to the left panel as indicated. The central leucin of the substrate peptide forms hydrophobic contacts with I438 on β-strand 4. This interaction is transmitted to V440 on strand four and further, through hydrophobic interactions, to L484 on β-strand 6. L484 forms hydrogen bond interactions with D148 that is connected through a rigid loop with P143. P143 contacts K70 that forms a hydrogen bond with the γ-phosphate of ATP and stabilizes the transition state of hydrolysis. In this way binding of substrates is directly transmitted into the catalytic center. **(D)**, Cartoon of ATP induced docking of SBDβ and NBD and substrate induced ATP hydrolysis and transition to the high affinity conformation of the SBDβ. SBDα is omitted for clarity. Indicated are ATP induced rotation of the NBD lobes and residues (D481→R167/I168; K414→D326) that form the clamp to prevent back rotation of the NBD lobes, as well as residues (I438, V440, L484, D148, P143, K70) that are important for transmission of the substrate binding signal to the catalytic center for γ-phosphate cleavage. The J-domain is important for tight coupling of substrate binding and signal transmission (more detailed in Figure 4).

Genetic screens and structural studies on the individual domains of DnaK revealed single residues that are important for the allosteric mechanism (Burkholder et al., 1994; Laufen et al., 1999; Montgomery et al., 1999; Vogel et al., 2006a; Vogel et al., 2006b; Smock et al., 2010; Kumar et al., 2011). A general feature of amino acid replacements outside the ATP binding pocket itself that disturb the allosteric regulation is an increased intrinsic ATPase activity (Figure 3A). It can be concluded from this observation that allosteric coupling of the NBD and SBD inhibits γ-phosphate cleavage in the NBD. Those amino acid replacements that have the largest impact on intrinsic ATPase rate indicate residues that are most important for inhibiting the ATPase activity. However, the isolated NBD has an ATPase activity as low as full-length DnaK, arguing against an inhibitory effect of the SBD. This conundrum was solved by the discovery that the highly conserved linker between NBD and SBD has an important impact on interdomain communication and on the intrinsic ATPase activity of the NBD as well. Prolonging the NBD with the linker residues [386VKDVLLLD393; DnaK(1–393)] increased the ATPase rate 40-fold (Vogel et al., 2006b; Swain et al., 2007; English et al., 2017) and this effect is abrogated or greatly diminished when the hydrophobic residues of the linker or D393 are replaced by alanine. Similar observations were also made for HscA (Alderson et al., 2014). These intriguing observations fall into place in the structure of DnaK in the ATP-bound open conformation that allowed to trace these residues complemented by additional residues into a network of hydrogen bonds and hydrophobic interactions that mediate interdomain communication and allosteric regulation (Kityk et al., 2012; Kityk et al., 2015) (Figures 3B–D–D). In general, these residues are highly conserved in Hsp70s from bacteria to humans and their presence is indicative for an allosteric mechanism. Albeit, some of the residues are conservatively replaced in some branches of the Hsp70 tree with consequences for the equilibrium between the different conformational states of Hsp70s (Zhuravleva et al., 2012).

Comparison of the crystal structures of Hsp70s in the ADP and ATP bound states revealed that upon ATP binding to Hsp70 the two lobes of the NBD rotate relative to each other and allow the SBDβ to dock onto the NBD. Two effects are responsible for the low ATP hydrolysis rates and thus the high enthalpy of activation of γ-phosphate cleavage. First, a single proline in the NBD (P143) stabilizes the ATP-bound state and upon replacement of this proline by glycine the enthalpy of activation for ATP hydrolysis decreases to 50% of the value for wild-type DnaK (Vogel et al., 2006a). Second, the SBDβ clamps down the rotated position of the NBD lobes resulting in a geometry of the catalytic residues in the ATP binding pocket that is unfit for ATP hydrolysis (Figure 3C). This clamp contributes some 30% to the enthalpy of activation as deduced from the difference in activation enthalpy for ATP hydrolysis for DnaKwt and DnaK(2–385) (Vogel et al., 2006a). The two residues in the SBDβ that contributes most to this clamping of the NBD are D481, interacting with the backbone of I168 in lobe I and K414, interacting with D326 in lobe II (Figures 3B,**C**). Replacement of D481 by alanine or K414 by isoleucine increases the intrinsic ATPase activity by 80-fold and 25-fold, respectively (Kityk et al., 2015). Binding of a polypeptide substrate to the substrate binding pocket triggers ATP hydrolysis by acting through a defined intramolecular signal transduction pathway involving V440 and L484 in the SBDβ and D148 in the NBD (Kityk et al., 2015) (Figures 3C,D). Replacement of any of these residues with alanine leads to a complete loss of substrate stimulation of the ATPase activity but not of the stimulation of the ATPase activity by DnaJ.

## Hsp70 Interaction With Cochaperones

### J-Domain Proteins: Hsp70 Targeting Factors

**FIGURE 4:**
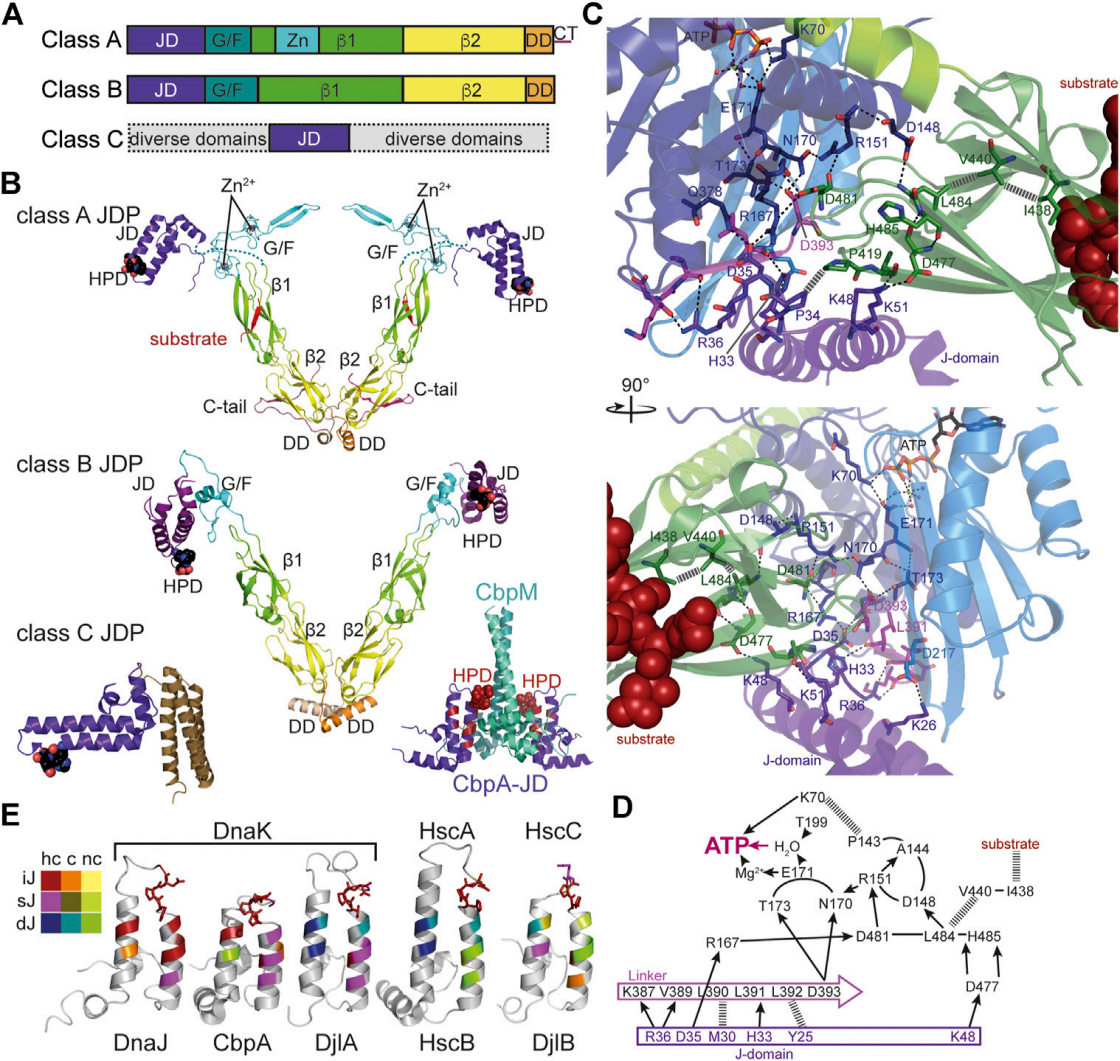
Structure and function of J-domain proteins (JDPs). **(A)**, Domain structure of the three classes of JDPs; JD, J-domain; G/F, glycine-phenylalanine rich region; β1/2, β-sandwich domain 1 and 2; Zn, Zn^2+^-finger domain; DD, dimerization domain; CT, C-terminal tail. **(B)**, Cartoon representation of the crystal structures of JDPs; domains colored as in A; HPD motif in space-filling representation. Top panel, structure of the *S. cerevisiae* class A JDP Ydj1 since no structure for a class A JDP of prokaryotic origin has been solved to my knowledge. Composite of crystal structures 1NLT (Li et al., 2003) and 1XAO (Wu et al., 2005) and the NMR structure of the J-domain 5VSO (Schilke et al., 2017). The location of the J-domain is arbitrary as it is connected to the β1-domain by the flexible G/F-rich region shown as dashes. Middle panel, crystal structure of the class B JDP DnaJ of *Thermus thermophilus* [4J80, (Barends et al., 2013)]. Inset to the lower right, Inhibitory complex between the CbpM dimer (greencyan and deepteal) and two J-domains of the class B JDP CbpA (purple; 3UCS). Residues homologous to DnaJ residues that interact with DnaK in the co-crystal structure are colored in dark red. HPD-motif shown as spheres. Bottom left panel, crystal structure of the class C JDP HscB of *E. coli* [1FPO, (Cupp-Vickery and Vickery, 2000)]. **(C)**, Zoom into the crystal structure of *E. coli* DnaK in complex with the J-domain of DnaJ [5NRO, (Kityk et al., 2018)], illustrating how the J-domain contacts the allosteric network of polar (black dashed lines) and non-polar (gray hatched lines) contacts connecting the substrate binding pocket with the catalytic center for ATP hydrolysis. Lower panel, rotated by 90° as compared to the upper panel as indicated. **(D)**, Schematic representation of the interaction network contacted by the J-domain. Arrows indicate polar contacts; hatched lines indicate non-polar interactions; other lines indicate peptide backbone connections. **(E)**, Structures of J-domains of DnaJ, CbpA, DjlA, HscB and DjlB colored according to conservation of residues interacting with DnaK in the co-crystal structure of DnaK and the J-domain of DnaJ [5NRO, (Kityk et al., 2018)]. NMR structures of J-domains of DnaJ [1XBL, (Pellecchia et al., 1996), and CbpA (2KQX, (Sarraf et al., 2010)]; crystal structure of the J-domain of HscB [1FPO, (Cupp-Vickery and Vickery, 2000)]; homology models of the J-domains of DjlA and DjlB using SWISS-Model (Bienert et al., 2017; Waterhouse et al., 2018). Color scheme indicated to the left; hc, highly conserved (>60% identity and >90% similarity in a CLUSTAL Ω alignment of 200 mutually less than 90% identical sequences; UniRef90 database), c, conserved (>80% similarity), nc, not conserved; iJ, identical as in *E. coli* DnaJ; sJ, similar as in *E. coli* DnaJ; dJ, different (non-conservative replacement) to the residue in *E. coli* DnaJ. HPD motif in stick representation.

JDPs are modular multi-domain proteins that are essential cochaperones of Hsp70s. Common to all JDPs is the so-called J-domain, an α-helical hair-pin domain of generally 70–75 residues in length, which is essential for triggering in synergism with protein substrates ATP hydrolysis in Hsp70s. The additional domains of JDPs allow them to interact with protein clients of Hsp70s or to be localized within the cell where Hsp70 clients appear, e.g., at the ribosome or at translocation pores. Their main function is to target Hsp70s to client proteins and trigger client trapping. JDPs are generally divided into three classes according to the number of domains they have in common with the prototype of JDPs, *E. coli* DnaJ (Kampinga and Craig, 2010) (Figures 4A,B). Class A JDPs are 360–400 amino acids long and have a domain architecture like DnaJ: an N-terminal J-domain followed by a glycine-phenylalanine rich region (G/F-region), two homologous β-sandwich domains with a zinc-finger inserted in the first of the two domains, and a C-terminal dimerization domain with an intrinsically disordered tail. Client binding sites are found in the β-sandwich domains (Jiang et al., 2019) and also the zinc-finger seems to be involved in substrate binding and prevention of aggregation activity (Linke et al., 2003). Class B JDPs are in general 260–360 amino acids long and differ from DnaJ by the lack of the zinc-finger and the C-terminal tail. Both, class A and class B JDPs are considered as general JDPs that are able to bind to essentially all partially folded, misfolded and aggregated proteins. Both classes seem to form V-shaped dimers with the protomers linked together through a flexible C-terminal hinge (Sha et al., 2000; Wu et al., 2005; Barends et al., 2013) (Figure 4B). Thus, they could bind simultaneously to at least two sites within misfolded polypeptide and aggregates, which might be an efficient way to distinguish native from non-native proteins. Class C JDPs are very heterogeneous with 54 to more than 1,000 amino acids in length and only share with DnaJ the J-domain that might be found anywhere within the sequence. They may contain a number of other domains, most notably specific protein-protein interaction domains, DNA and RNA binding domains, and transmembrane regions. An extensive analysis of JDP associated domains in prokaryotes can be found in (Barriot et al., 2020). In some cases, it seems that the J-domain was an add-on late in evolution to make cellular processes more efficient by providing chaperone power (Sahi et al., 2010). *E. coli* contains one class A (DnaJ), one class B (CbpA) and four class C JDPs (HscB, DjlA, DjlB, and DjlC), whereby DnaJ, CbpA, and DjlA functionally interact with DnaK; HscB with HscA (Silberg et al., 1998); and DjlB and DjlC with HscC (Kluck et al., 2002) (Figure 4E).

How the J-domain stimulates ATP hydrolysis was recently elucidated by crystallization of the J-domain of *E. coli* DnaJ in complex with DnaK in the ATP bound state (Kityk et al., 2018) (Figure 4C). The J-domain binds on top of the interdomain linker that is important for the stimulation of the ATPase activity and interacts with NBD and SBDβ (Vogel et al., 2006b; Swain et al., 2007). It is positioned by electrostatic interaction between positively charged residues in the J-domain (R22, K26, R27, K48, K51) and negatively charged residues in the NBD (E206, D211, E217) and SBDβ (D477) as had been proposed based on NMR and computational data (Ahmad et al., 2011; Malinverni et al., 2017; Tomiczek et al., 2020). Genetic screens had identified the highly conserved histidine-proline-aspartate (HPD) motif as essential for the functional interaction of the J-domain with Hsp70. The replacement of histidine or aspartate within this motif for glutamine or asparagine, respectively, abrogated the ability of the J-domain to stimulate the ATPase activity of Hsp70s in every system tested so far [e.g., (Wall et al., 1994; Tsai and Douglas, 1996; Kelley and Georgopoulos, 1997; Liu et al., 1998; Chevalier et al., 2000; Morgan et al., 2001; Mokranjac et al., 2003)]. H33 of the DnaJ HPD motif forms a hydrogen bond with the backbone carbonyl of L391 of the interdomain linker of DnaK, P34 forms hydrophobic contacts to P419 in the SBDβ of DnaK, and D35 forms hydrogen bonds to R167 and Q378 of DnaK. L391 had previously been implicated in allosteric regulation (Kumar et al., 2011) and R167 in interaction with DnaJ (Suh et al., 1998). The J-domain interacts directly with the network of hydrogen bonds that converge in two branches onto the γ-phosphate of the ATP (Figures 4C,D). Intriguing was the finding that the J-domain contacts the SBDβ through a salt bridge (J-domain K48→DnaK-D477) and thereby seems to stabilize the signal transduction pathway that transmits the signal of the bound client to the NBD for triggering ATP hydrolysis (Kityk et al., 2018). The residues of the J-domain that interact with DnaK are well conserved in JDPs known to interact with a DnaK-type Hsp70 (Figure 4E), explaining the promiscuity of J-domains as demonstrated by grafting J-domains from JDPs of a wide variety of organisms onto *E. coli* DnaJ to study their functionality [e.g., (Kelley and Georgopoulos, 1997; Nicoll et al., 2007; Maillot et al., 2019)]. However, there is also specificity as some of the residues of the J-domain that interact in the crystal structure with DnaK are different in specific subgroups of JDPs in particular those that do not interact with DnaK and well conserved within the respective JDP subfamily as sequence alignments revealed (Figure 4E). The functional significance of these differences has not been analyzed in detail and it is currently not known, which of the differences are the result of coevolution of functional Hsp70-JDP pairs and which are the result of phylogenetic relationship. An extensive phylogenetic analysis of prokaryotic JDPs was recently published (Barriot et al., 2020).

A recent NMR study elucidated that class A and class B JDPs bind polypeptides in a highly dynamic multivalent manner using up to four low-affinity sites, one in each of the four β-sandwich domains of the JDP-dimer (Jiang et al., 2019). This explains the earlier observation that JDPs generally bind peptides with much lower affinity than protein clients (Rüdiger et al., 2001). Such a binding mode has two consequences. First, JDPs only bind proteins stably when a sufficient number of binding sites for the JDP are exposed, which is generally only the case in the nascent, not yet folded, and the misfolded state. The more binding sites are exposed within a polypeptide in a suitable geometry, the higher the overall affinity of JDPs to the client due to the avidity effect. This also explains why the human class B JDP DnaJB1 distinguishes α-synuclein amyloid fibrils from the intrinsically disordered monomer: at least two binding sites in neighboring α-synuclein protomers within the amyloid fibril are necessary for high affinity interaction (Gao et al., 2015; Wentink et al., 2020). Second, such a binding mode allows for rapid association and dissociation of individual binding sites from the JDP favoring an efficient transfer of the client onto Hsp70s. The NMR investigation further revealed that JDPs mainly interact with amino acid sidechains and not with the backbone (Jiang et al., 2019), consistent with a binding mode that was proposed earlier based on peptide library scanning (Rüdiger et al., 2001) and with hydrogen exchange mass spectrometry data (Rodriguez et al., 2008).

Interestingly, the yeast class A JDP Ydj1 sports an intrinsically disordered C-terminal tail that binds to the substrate binding site in the second β-sandwich domain and seems to compete with client binding (Wu et al., 2005; Jiang et al., 2019) (Figure 4B). Similar disordered tails are also found in prokaryotic class A JDPs as multiple sequence alignments reveal. A competition of the C-terminal tail with substrates for binding to the second β-sandwich domain might limit the overall affinity of JDPs to very hydrophobic substrates by autoinhibition to prevent quasi irreversible binding. It also might facilitate client transfer onto Hsp70s or release of the JDP from the substrate polypeptide after transfer of a single binding site to Hsp70. Such an autoinhibitory C-terminal tail is missing in class B JDPs. Intriguingly, eukaryotic class B JDPs seem to be self-inhibited in a different way by a small α-helix in the G/F-region that binds to the J-domain and apparently blocks its interaction with Hsp70 (Faust et al., 2020). This block is relieved by binding of the EEVD motif to the first β-sandwich domain (Li et al., 2006; Yu et al., 2015; Faust et al., 2020). The molecular mechanism of how binding of the EEVD motif unlocks the J-domain of class B JDPs is still a mystery. When the EEVD motif at the C-terminus of the eukaryotic Hsp70 is deleted it still can refold a misfolded model substrate in cooperation with the class A JDP but not anymore with a class B JDP. Whether such an inhibitory mechanism also exists in prokaryotic class B JDPs is currently unknown. The sequence of prokaryotic Hsp70s generally does not end in an EEVD motif. However, many DnaK-type prokaryotic Hsp70s contain a glutamate and aspartate rich sequence close to the very C-terminus and deletion of the last seven residues including an EEV sequence in *E. coli* DnaK reduces its ability to complement the temperature sensitivity phenotype of a *∆dnaK* strain (Smock et al., 2011). Furthermore, the crystal structure of the class B JDP of *Thermus thermophilus* revealed an α-helix within the G/F-region that was docked onto the J-domain (Figure 4B) (Barends et al., 2013). Furthermore, CbpA is inhibited *in vitro* and *in vivo* by a small protein CbpM that is encoded in the same operon downstream of *cbpA* in *E. coli* and conserved in γ-proteobacteria (Chae et al., 2004; Chenoweth et al., 2007). CbpM is specific for CbpA and does not inhibit the interaction of DnaJ with DnaK. CbpM binds to the J-domain of CbpA in a way that blocks access to DnaK (Sarraf et al., 2014) (Figure 4B lower right panel). Overexpression of CbpM in the background of a *∆dnaJ* strain phenocopies a *∆dnaJ ∆cbpA* strain. Why the inhibition of the CbpA-DnaK interaction is advantageous is not clear.

In eukaryotic Hsp70 systems JDPs of class A and class B cooperate in protein disaggregation (Nillegoda et al., 2015). However, this does not seem to be the case for prokaryotic JDPs (Nillegoda et al., 2017).

### Nucleotide Exchange Factors: Timing the Hsp70-Client Interaction

Since at physiological ATP concentrations nucleotide exchange is rate-limiting for release of bound polypeptide clients, NEFs regulate the lifetime of the Hsp70-client complex. Currently, four evolutionarily unrelated families of NEFs for Hsp70s are known that use different mechanisms to open the nucleotide binding cleft of Hsp70s and thereby to accelerate nucleotide dissociation. Three of the four families of NEFs are only found in eukaryotic cells and are not further discussed here [for detailed discussion see (Bracher et al., 2015; Mayer and Gierasch, 2019)].

**FIGURE 5:**
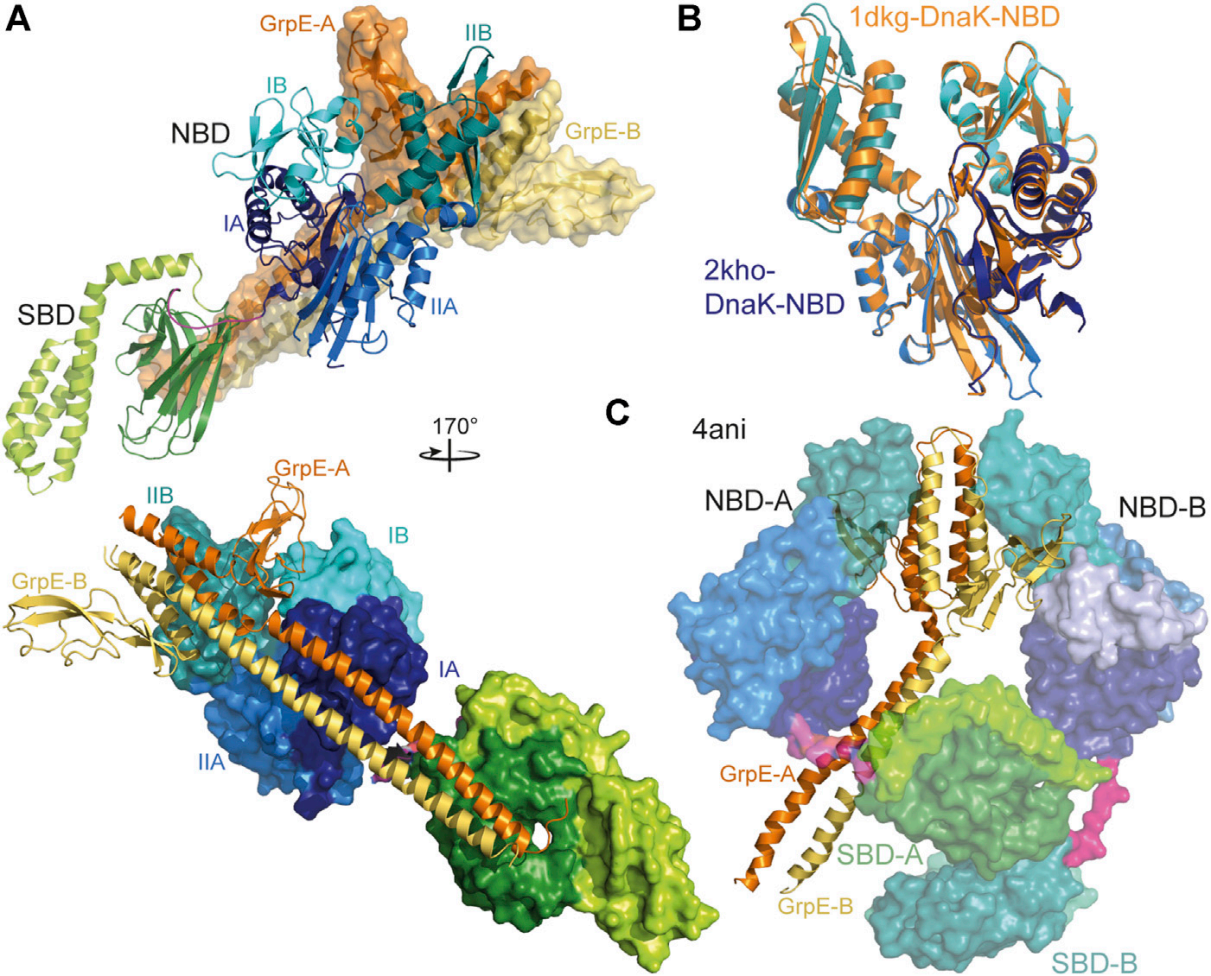
Interaction of GrpE with DnaK. **(A)**, overlay of the crystal structure of *E. coli* GrpE in complex with the NBD of *E. coli* DnaK [1DKG, (Harrison et al., 1997)] onto the solution structure of *E. coli* DnaK in the ADP-bound conformation [2KHO, (Bertelsen et al., 2009)]. Top panel, DnaK as cartoon, GrpE in surface representation. Bottom panel, rotated by 170° as compared to the top panel and DnaK in surface representation and GrpE as cartoon. **(B)**, overlay of the NBD of *E. coli* DnaK in the absence (colored different shades of blue according to subdomains) and presence (orange) of GrpE indicating the 14° outward tilt of subdomain IIB. **(C)**, crystal structure of two molecules of *Geobacillus kaustophilus* DnaK (surface representation) in complex with the GrpE dimer (cartoon).

In prokaryotes, mitochondria and chloroplasts nucleotide exchange in Hsp70s is stimulated by GrpE, a homodimeric protein that consists of an N-terminal intrinsically disordered region of some 40 residues followed by an unusually long α-helical dimerization domain and a C-terminal β-sheet domain. GrpE interacts with DnaK in an asymmetric 2-to-1 complex, inserting the β-sheet domain into the nucleotide binding cleft and opening it by tilting subdomain IIB by 14° outward (Harrison et al., 1997) (Figures 5A,B). In contrast, *Geobacillus kaustophilus* GrpE and DnaK crystallized in a GrpE_2_·DnaK_2_ complex that was nevertheless asymmetric (Wu et al., 2012) (Figure 5C). So far there is no evidence that this structure represents a functional state that also exists in other prokaryotic organisms, and that GrpE in this way triggers nucleotide exchange and thus client release by two Hsp70 chaperones in a coordinated fashion. GrpE was also proposed to induce polypeptide client release in the absence of ATP. This hypothesis was based on the position of GrpE in the crystal structure suggesting that the intrinsically disordered region at the N-terminus of GrpE, which is well conserved in length within prokaryotic GrpE homologues, might be close to the polypeptide binding groove of Hsp70 (Figures 5A,B). However, careful analysis revealed that GrpE does not accelerate client dissociation but prevents rebinding by competing with its N-terminal tail for the client binding groove (Brehmer et al., 2004). GrpE might thus act in a similar way as was recently shown for the HspBP1 NEF in eukaryotic cells (Gowda et al., 2018).

Some Hsp70s do not seem to need a NEF since they have a very high intrinsic ADP dissociation rate (Brehmer et al., 2001). This raises the question why NEFs are needed at all, since ADP dissociation rates could be tuned to the optimal value. Such an optimal tuning might be advantageous for Hsp70s that interact with one or a small number of defined clients, but not for Hsp70s that are generalists and interact with a wide variety of clients that need different residence times on Hsp70. Maybe stochastic interaction of GrpE with DnaK will yield at least in a fraction of the cycles the exact optimal lifetime of the DnaK-client complex. Another advantage of NEFs could be localized nucleotide exchange. Some eukaryotic NEFs are targeted to specific locations within the cell, for example the ER or plasma membrane, and for these NEFs it seems plausible that nucleotide exchange and therefore release of client from Hsp70s occurs at specific subcellular sites. In contrast, GrpE in *E. coli* is homogenously distributed throughout the cell at optimal growth conditions, as well as, during heat shock (Kumar and Sourjik, 2012), refuting such a hypothesis for GrpE. Alternatively, NEFs could link nucleotide exchange and thereby polypeptide release to environmental conditions. At heat shock temperatures above 42°C for *E. coli* or 85°C for *Thermus thermophilus* GrpE starts to unfold reversibly and becomes inactive (Grimshaw et al., 2001; Groemping and Reinstein, 2001; Grimshaw et al., 2003). Such an unfolding would slow down nucleotide exchange and client release from Hsp70 under condition when reaching the native state is unlikely. Upon return to normal growth temperatures GrpE refolds and becomes active again.

## Hsp70 Interaction With Substrates

A proteomic study showed that DnaK in *E. coli* interacts with at least 700 proteins among which are some 180 aggregation-prone proteins that remained bound to DnaK for an extended period of time (Calloni et al., 2012). This number increases to some 1,000 proteins in *E. coli* cells deleted for the ribosome-associated chaperone trigger factor. In fact, it was shown that the DnaK-DnaJ-GrpE team can keep proteins in an active state under conditions when the thermodynamic equilibrium would drive the protein into the denatured state (Goloubinoff et al., 2018). Thus, DnaK uses ATP to continuously drive the protein out of thermodynamic equilibrium.

At 37°C most proteins are bound transiently by DnaK in the nascent state during synthesis at the ribosome. This observation is well explained by peptide library scanning data (Rüdiger et al., 1997) that revealed the recognition motif for DnaK binding. This motif consists of a core of five residues enriched in hydrophobic amino acids with a strong preference for leucine, flanked by regions enriched in positively charged residues. Negatively charged residues disfavor DnaK binding. Such motifs are found on average every 30–40 residues in practically all proteins except for intrinsically disordered proteins that are generally depleted of hydrophobic amino acids. In the structure of most proteins the DnaK binding motifs are found in the hydrophobic core and only accessible in the nascent and denatured state.

**FIGURE 6:**
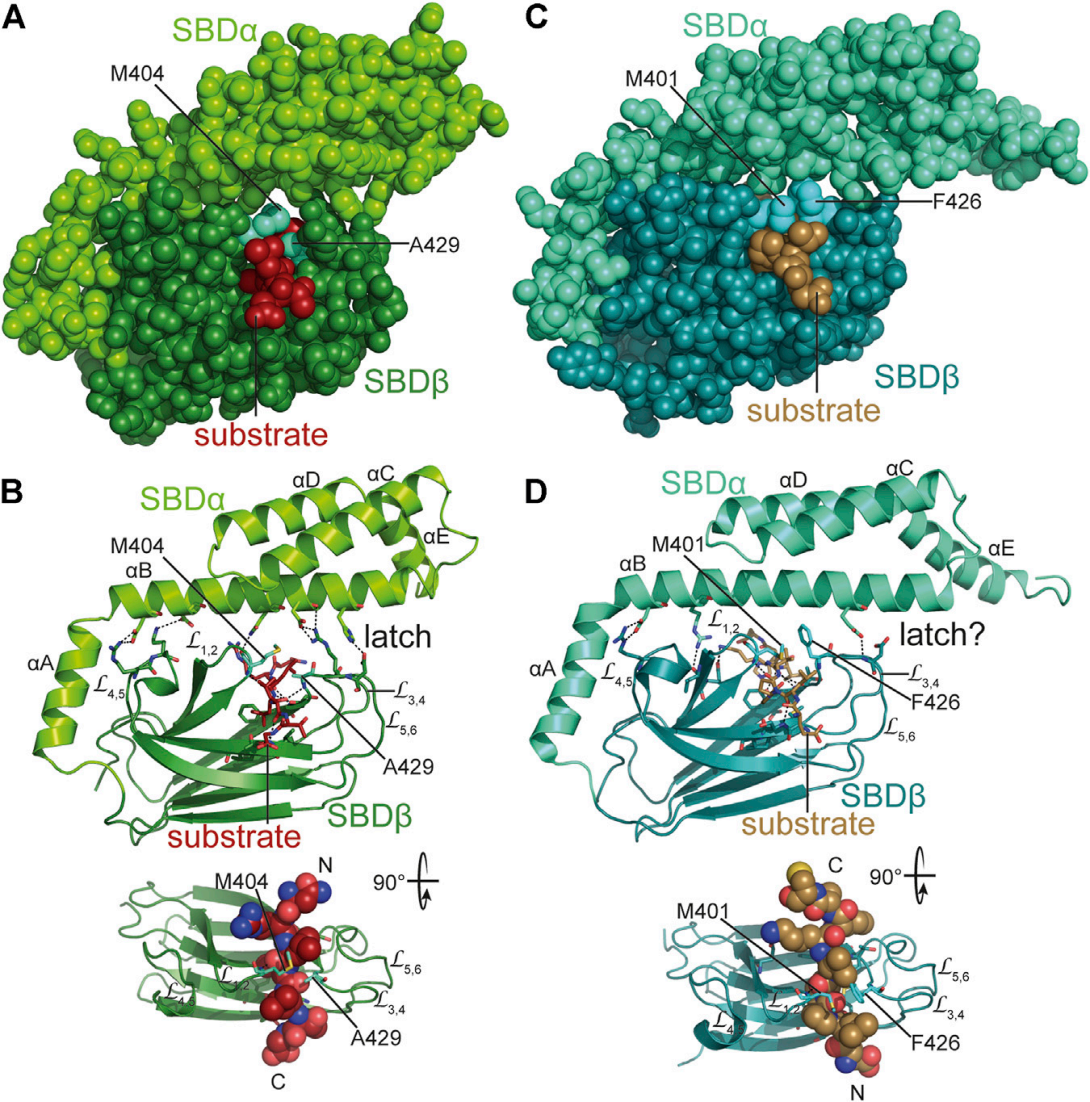
Interaction of Hsp70s with peptide substrates. **(A,B)**, crystal structure of *E. coli* DnaK SBD in complex with a peptide substrate (NRLLLTG) in space-filling representation **(A)** and as cartoon **(B)** [1DKX, (Zhu et al., 1996)]. Polar contacts between SBDβ and SBDα, as well as, between SBDβ and substrate peptide are shown as black dashed lines. Arch forming residues M404 and A429 are labeled. Lower panel, SBDβ rotated as indicated with substrate peptide in space-filling representation and N-and C-terminus of the bound peptide labeled with N and C. **(C,D)**, crystal structure of the SBD of *E. coli* HscA in complex with a peptide (ELPPVKIHC) comprising the interaction sequence in IscU [1U00, (Cupp-Vickery et al., 2004)] in space-filling **(C)** and cartoon **(D)** representation. Arch forming residues M401 and F426 are labeled. Whether the single hydrogen bond between SBDα and outer loops of SBDβ functions as a latch is unclear. Lower panel, SBDβ rotated as indicated with substrate peptide in space-filling representation and N-and C-terminus of the bound peptide labeled with N and C.

The crystal structures of the SBD of DnaK in complex with different substrate peptides (Zhu et al., 1996; Zahn et al., 2013) show the peptide well engulfed by the upward protruding loops forming hydrophobic contacts with the sidechains of the peptide and hydrogen bonds with the peptide backbone (Figures 6A,B). Therefore, DnaK in contrast to DnaJ distinguishes well between peptides made from L-and D-amino acids (Rüdiger et al., 2001). The SBDα lid forming a latch of a salt bridge and hydrogen bonds with the outer loop contributes to the affinity of DnaK to its substrate peptide decreasing peptide dissociation rates substantially (Mayer et al., 2000; Moro et al., 2004). However, electron paramagnetic resonance spectroscopy and Förster resonance energy transfer (FRET) measurements revealed that the lid does not necessarily close entirely over bound protein clients (Marcinowski et al., 2011; Schlecht et al., 2011). In fact, optical tweezer experiments showed that DnaK can bind to folding intermediates preventing their unfolding against external pulling force (Mashaghi et al., 2016). For the latter binding mode, the lid was more important than the peptide binding groove as an amino acid replacement that lowered the affinity for peptide binding to 1/40th of the wild type affinity (K_D_ 40-fold increased), had little effect on the force induced unfolding of the client protein, whereas a truncation of the lid in the middle of helix B abrogated the ability of DnaK to counteract force induced client unfolding. Therefore, the picture that the crystal structures convey may be representative for Hsp70 binding to nascent polypeptide chains but not so much for interaction of Hsp70 with folding intermediates or misfolded proteins.

Moreover, the C-terminal intrinsically disordered region also seems to contribute to the interaction with client proteins as C-terminally truncated DnaKs are less efficient in complementing the temperature sensitivity phenotype of a *∆dnaK* strain than wild type DnaK and also less efficiently assists refolding of denatured model clients *in vitro* (Smock et al., 2011). In addition, electron paramagnetic resonance spectroscopy using a nitroxide label in the C-terminal tail indicates high mobility of the tail in the absence of a client protein or in the presence of short peptide substrate but restricted mobility in the presence of a misfolded protein client, suggesting direct interaction of the tail with the misfolded client protein.

How are Hsp70s able to refold denatured inactive proteins to the native active state? Several studies suggest that the major action of Hsp70s is local unfolding. Sharma and colleagues found that thioflavin T fluorescence and protease resistance of a misfolded protein client decrease upon addition of DnaK, DnaJ, GrpE and ATP (Sharma et al., 2010), indicating unfolding of the client. NMR experiments with a small single domain protein indicated that DnaK binds to a transiently unfolding state of the protein (conformational selection) and keeps the protein in a state devoid of tertiary structure but that still contained secondary structure distal of the DnaK binding site (Sekhar et al., 2015; Sekhar et al., 2016; Sekhar et al., 2018). Interestingly, in this case the conformation of the client protein was independent of the nucleotide state of DnaK, suggesting that DnaK did not alter the conformation of the client in the binding process. However, DnaK could bind to four different binding sites within the 53-residue client protein and in some cases two DnaK molecules could bind to the same client molecule (Rosenzweig et al., 2017). Single molecule FRET measurements monitored a large expansion of a protein in the presence of DnaJ and DnaK and ATP (Kellner et al., 2014). Albeit, it should be noted that rhodanese the model substrate used in this study cannot be refolded by the DnaK-DnaJ-GrpE chaperone team but requires the GroEL-GroES machinery (Mendoza et al., 1991; Mayhew et al., 1996). A more recent hydrogen exchange mass spectrometry and single molecule FRET study using luciferase as DnaK model client revealed also extensive unfolding by DnaK (Imamoglu et al., 2020). The unfolding was achieved by binding of several DnaK molecules to a single misfolded client protein. It could be imagined that at an initial stage DnaK binds to an exposed site and prevents this hydrophobic region to associate with similar regions to form aggregates. As the bound client protein undergoes thermal movements additional sites are exposed that then can be bound by DnaK. Alternatively or in addition, DnaJ may play a more active role in the unfolding process. Since DnaJ interacts with sidechains of hydrophobic amino acids and does not need the peptide backbone for interaction, it could scan the surface of misfolded proteins for regions prone to aggregation. DnaJ was also shown to induce partial unfolding (Rodriguez et al., 2008; Kellner et al., 2014) that could favor exposure of DnaK binding sites. Furthermore, entropic pulling was introduced as mode of action for Hsp70- mediated force exertion. Originally this concept was introduced to explain import of polypeptides into the mitochondrial matrix and for solubilization of protein aggregates (De Los Rios et al., 2006). Briefly, translocating polypeptide chains that reach the mitochondrial matrix through the Tim23 import pore are bound close to the membrane by the matrix resident Hsp70. Since Hsp70 constitutes a bulky entity that restricts the conformational freedom of the incoming polypeptide this state has a low entropy and entropy increases as the Hsp70 moves away from the membrane taking the bound polypeptide with it. As chemical reaction can be driven by increasing entropy, this mechanism leads to import of the polypeptides and exerts a considerable force on the polypeptide, driving unfolding of the transport protein on the other side of the membrane. The entropic pulling force decreases with increasing polypeptide length translocated into the matrix and reaches zero at a translocated length of about 30 residues, whereupon a second Hsp70 has to bind the incoming chain close to the membrane. Experimental proof for such a mode of action was recently achieved for the disassembly of trimeric human heat shock transcription factor (Kmiecik et al., 2020). Hsp70s bind close to the trimerization domain and monomerize Hsf1 trimers. If the Hsp70 binding site is moved away from the trimerization domain along an intrinsically disordered region, Hsf1 monomerization occurs at lower rates and cease when the binding site is 20 or more residues away from the trimerization domain. Binding of several Hsp70 to a single Hsf1 trimer accelerates monomerization, providing additional evidence for entropic pulling as physical principal for the reaction, as local crowding would be expected to increase the entropic pulling force. Local crowding also seems to drive Hsp70 action in clathrin uncoating (Sousa et al., 2016) and in the fragmentation of α-synuclein fibrils (Wentink et al., 2020). It was also suggested that Hsp70s facilitate the sliding of nascent chains through the ribosomal exit tunnel by entropic pulling. Translation elongation pauses under conditions in which Hsp70 activity is limiting, as during heat shock, sever proteotoxic stress, or upon expression of a dominant negative Hsp70. Such a pausing is not observed when intracellular Hsp70 concentrations are increased prior to stress exposure (Liu et al., 2013; Shalgi et al., 2013). Moreover, ribosomal profiling revealed that translation speed increases when the yeast Hsp70 Ssb1 binds to the nascent chain which would be consistent with Ssb1 speeding-up translation by facilitating the sliding of the nascent chain through the ribosomal exit tunnel by entropic pulling (Döring et al., 2017). Similarly, entropic pulling could lead to stepwise unfolding of a misfolded protein when several Hsp70s and a JDP bind to the protein creating local crowding and a state of low entropy.

At physiologically high concentrations of DnaK (15–20 µM), the association rates of new DnaK molecules binding to the client might be higher than the dissociation rate of already bound DnaK molecules preventing folding to proceed and causing a deadlock. Such a deadlock can be resolved by the Hsp90 chaperone HtpG of *E. coli* (Morán Luengo et al., 2018). This cooperation between Hsp70 and Hsp90 chaperones is also found in eukaryotic cells and does not require the Hsp70-Hsp90 organizing protein Hop (Bhattacharya et al., 2020).

## Hsp70 Complexes–A New Mode of Hsp70 Action?

Hsp70 oligomerization/polymerization in the nucleotide-free or ADP bound state has been known for a number of years (Schmid et al., 1985; Freiden et al., 1992; Blond-Elguindi et al., 1993; Benaroudj et al., 1995; King et al., 1995; Schönfeld et al., 1995; Angelidis et al., 1999; Thompson et al., 2012; Preissler et al., 2015). More recently, based on the dimer assembly in crystal structures Hsp70-dimerization was proposed to also occur in the ATP bound state (Sarbeng et al., 2015).

**FIGURE 7:**
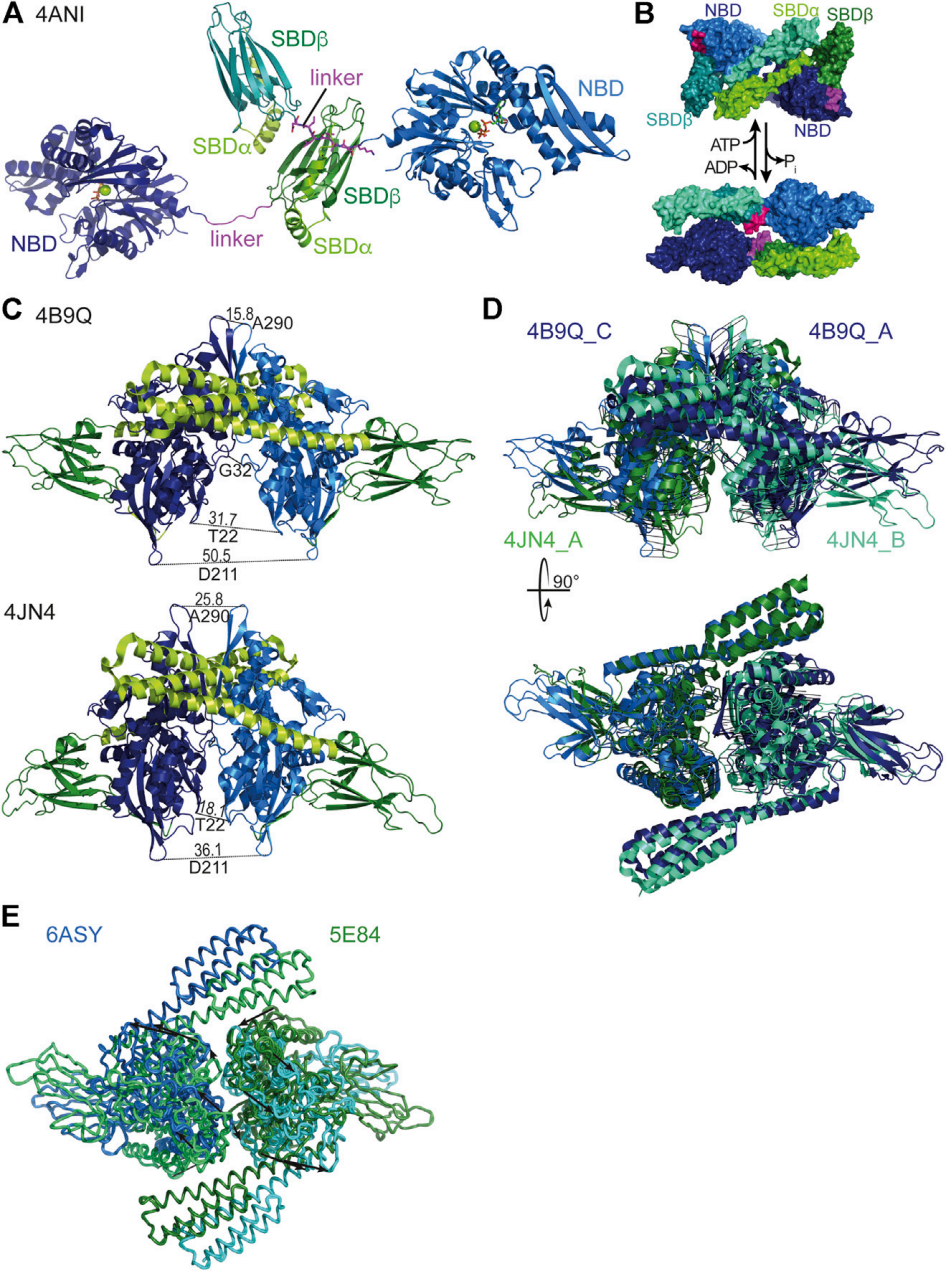
Oligomeric states of Hsp70s. **(A)**, dimeric assembly of *G. kaustophilus* DnaK with SBDβ of one DnaK molecule binding to the linker (magenta) of a second DnaK molecule [4ANI, (Chang et al., 2008a)]. **(B)**, Hsp70·ATP SBDα-SBDα dimeric assembly (4B9Q top) and Hsp70·ADP head-to-tail assembly (2KHO, bottom) as proposed based on native mass spectrometry and cross-linking (Morgner et al., 2015). **(C)**, DnaK·ATP dimers as found in the crystal structures [top, 4B9Q (Kityk et al., 2012); bottom, 4JN4 (Qi et al., 2013)]. To illustrate the differences the distances between identical residues in the two protomers are indicated. **(D)**, Overlay of the two crystal structures of DnaK·ATP by pairwise alignment of all residues in the NBDs (4B9Q chain A (dark blue) and C (light blue) to 4JN4 chain A (green cyan) and B (dark green); RMS = 4.573 over 747 residues). Black lines connect corresponding residues. Lower panel, same overlay rotated by 90° as indicated. **(E)**, Overlay of the two crystal structure of human BiP [5E84, (Yang et al., 2015a), and 6ASY, (Yang et al., 2017)]. Arrows connect corresponding residues and indicate the relative rotation of the protomers in the different dimer assemblies.

Hsp70 oligomerization in the ADP bound state or upon ATP hydrolysis has been suggested to be substrate-like binding of Hsp70 to itself based on the fact that 1) ATP converts the oligomer into monomers, which is analog to substrate release (Schmid et al., 1985); 2) substrates could compete with oligomerization (Freiden et al., 1992; Angelidis et al., 1999); 3) JDPs catalyze this type of interaction similar to substrate trapping (King et al., 1995). 4) Mutations that abrogate ATPase activity or decrease the affinity for substrates reduce oligomerization tendency (Thompson et al., 2012; Preissler et al., 2015). More precisely, crystallographic and biochemical data suggest that the SBD of one Hsp70 binds to the highly conserved hydrophobic NBD-SBD linker (KDVLLLD) of a second Hsp70 molecule (Chang et al., 2008a; Preissler et al., 2015; Preissler et al., 2020) (Figure 7A).

Such a mode of interaction would have the consequence that the Hsp70 engaged with the linker of another Hsp70 molecule would not be able to bind clients. Thus, oligomerization could be a mean for inactivation of Hsp70s when they are in unwanted excess. This function was proposed to neutralize excess of the endoplasmic reticulum Hsp70 BiP in the wake of the unfolded protein response. A dynamic monomer-oligomer equilibrium could rapidly adapt the amount of active BiP to fluctuations in unfolded protein load (Preissler et al., 2015).

A different type of Hsp70 dimer was recently proposed based on cross-linking and native mass-spectrometry data. This dimer, which is also believed to be promoted by JDPs, is envisioned to contain the two Hsp70 molecules in an anti-parallel arrangement, with the SBD of one Hsp70 being close to the NBD of the other, without engaging the interdomain linker like a substrate (Morgner et al., 2015) (Figure 7B). Most of the data provided in this publication are also consistent with the substrate-type oligomerization model described above. An exception is that an Hsp70 variant predicted to have a lower affinity for clients forms dimers to a similar extend as the wild-type protein, arguing for a different type of interaction. It was proposed that such an arrangement aids the loading of a native client onto Hsp90, albeit without supporting evidence.

A third type of Hsp70 oligomer is the NBD-NBD face-to-face dimer found in the crystal structures of the ATP bound open conformation of *E. coli* DnaK (Figure 7C) (Kityk et al., 2012; Qi et al., 2013). Interestingly, the dimer assembly in the two structures is not identical deviating in the tilt of the NBDs relative to each other by about 20° and rotated by about 30° (Figures 7C,D), suggesting a certain degree of flexibility in the assembly. However, in both structures the dimer interface covers a relatively large area (4JN4 1557 Å^2^, 4B9Q 1630 Å^2^), suggesting that this interface also exists in solution. Support for such an interface also comes from a Direct Coupling Analysis that found among 624 pairs of evolutionarily coupled residues six pairs for which a direct interaction would only be conceivable across the dimer interface (Malinverni et al., 2015). Analytical ultracentrifugation and cross-linking experiments suggest that about 14% of DnaK forms a dimer in solution at 15 µM concentration and about 3% at 4 μM, suggesting a K_D_ of 150–250 µM (Sarbeng et al., 2015). Amino acid replacements in DnaK that reduced the propensity for dimerization in the ATP bound state, without apparent defects in intrinsic ATPase activity, peptide binding or ATP-triggered conformational changes, showed reduced DnaJ-mediated substrate binding as measured by surface plasmon resonance (Mayer et al., 1999; Sarbeng et al., 2015), needed higher concentrations of DnaJ for refolding heat denatured luciferase *in vitro*, and some of the variants complemented the temperature sensitivity phenotype of a *∆dnaK E. coli* strain less well than wild-type DnaK.

What could be a possible advantage of the dimeric ATP bound state? Many JDPs are dimers in solution and in principle able to stimulate both Hsp70 molecules in the dimer assembly simultaneously, as the binding site for the J-domain is accessible in each protomer and the only structure of a full-length JDP has the J-domains at a sufficiently wide distance. In addition, JDPs may interact directly with Hsp70 substrates with several different interaction sites to present the substrate to the Hsp70 dimer. Simultaneous binding of both Hsp70s within the ATP-dimer seems possible, if the Hsp70 binding sites in the substrate polypeptide are more than 30 residues apart spanning in an extended conformation the distance of about 110 Å between the two substrate binding grooves in the Hsp70 dimer assembly (Figure 7C). This would fit the average 30 to 40 residues distance of good DnaK binding sites in proteins (Rüdiger et al., 1997). Such a binding mode seems advantageous for unfolding client proteins by the entropic pulling force of the two Hsp70 molecules that would detach from each other upon ATP hydrolysis.

## HscA and HscC: Variations of the Theme

**FIGURE 8:**
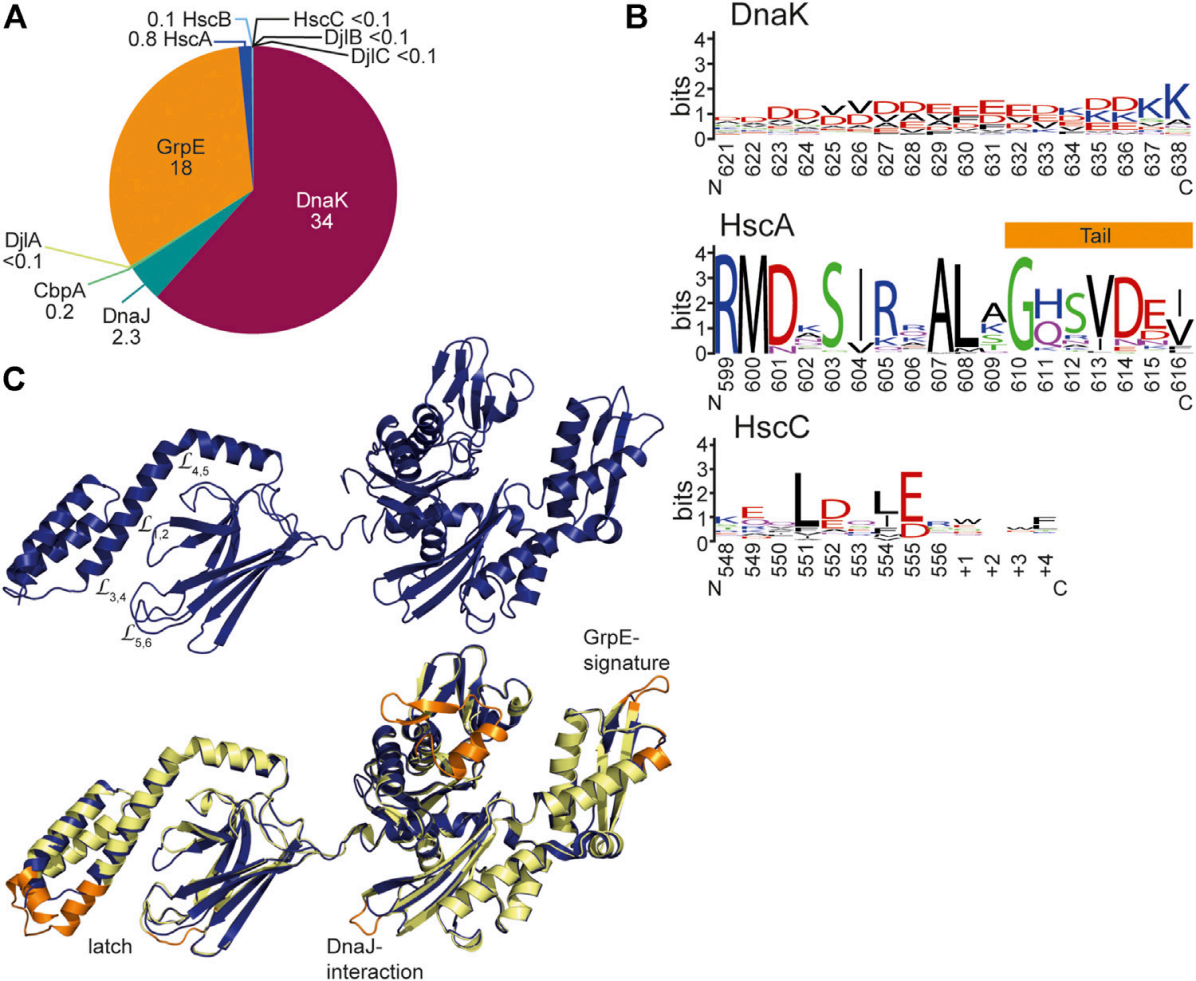
Structural differences between DnaK and HscA and HscC. **(A)**, Concentrations of the components of the Hsp70 systems in wild type *E. coli* in non-stress conditions and exponential growth phase. Numbers indicate concentrations in µM according to (Fauvet et al., 2021). <0.1 indicates that these components were below the detection limit of this quantitative mass spectrometry experiment. **(B)**, Weblogo of the C-terminal residues of DnaK, HscA and HscC. DnaK Weblogo, *E. coli* DnaK was used in a BLAST search against the UniRef90 database of clusters of mutually more than 90% identical sequences, and the C-terminal 18 residues of these representative sequences were used to generate the WebLogo since the C-terminal tail sequences do not align well in multiple sequence alignments due to low complexity (https://weblogo.berkeley.edu/logo.cgi) (Crooks et al., 2004). HscA Weblogo, 194 representative HscA sequences of the UniRef90 database were aligned using CLUSTAL Ω. The 18 residues that correspond to the unstructured tail in DnaK were used to generate the WebLogo. From the crystal structure of HscA-SBD helix E is longer in HscA than in DnaK and only the last 7 residues are unstructured. HscC WebLogo, 191 representative HscC sequences from the UniRef90 database were aligned using CLUSTAL Ω. The 13 residues (9 for *E. coli* HscC) that correspond to the C-terminal tail of DnaK were used to generate the WebLogo. **(C)**, Homology model of *E. coli* HscC generated using iTASSER (Yang et al., 2015b; Yang and Zhang, 2015). Lower panel, overlay of the homology model of HscC (dark blue) onto the solution conformation of *E. coli* DnaK (2KHO, (Bertelsen et al., 2009), shown in light yellow and orange. Orange are the sequence regions that are deleted in HscC.

In the exponential growth phase at optimal growth temperatures HscA and HscC and their JDP cochaperones are much less abundant in *E. coli* than the DnaK system. According to a recent quantitative proteomics study, DnaK constitutes under non-stress conditions 98% (ca. 34 µM) of all Hsp70 proteins and HscA 2% (0.8 µM), whereas HscC was below the detection limit (<0.1 µM) (Fauvet et al., 2021) (Figure 8A). GrpE (ca 18 µM) is about half as abundant as DnaK resulting in a stoichiometry of one GrpE dimer per four DnaK molecules. JDPs are much less abundant (DnaJ, 2.3 µM, CbpA, 0.2 µM, HscB, 0.1 µM and DjlA, DjlB, and DjlC below the detection limit in exponential growth phase) consistent with the catalytic function of the JDPs (Liberek et al., 1995).

The general structural organization of HscA and HscC-type Hsp70s is similar to the DnaK-type Hsp70s and most residues involved in ATP binding and hydrolysis as well as residues involved in allostery are either identical or replaced conservatively. It is therefore expected that the general working of these Hsp70 variants is similar to DnaK. However, there are a few distinctive features of HscAs and HscCs. Whereas the total length of DnaK-type prokaryotic and eukaryotic Hsp70s is around 640 residues (not counting signal sequences of the Hsp70s of mitochondria, plastids and endoplasmic reticulum), HscAs are generally 600–620 residues long and HscCs between 550 and 580 amino acids.

Most HscA proteins have an N-terminal extension of some 17 residues, the mechanistic significance of which is unclear. In HscAs the so-called GrpE-signature motif is deleted (residues 290–295) (Brehmer et al., 2001) and residues in DnaK that interact with GrpE are not conserved, suggesting that they do not interact with the NEF GrpE. For *E. coli* HscA it was shown that it has a 700-fold higher ADP dissociation rate as compared to DnaK. The reason for this increased nucleotide dissociation rate was found to be two salt bridges that bridge the nucleotide binding cleft in DnaK (K55-E267, R56-E264) but that are absent in HscAs. Further characteristic differences between HscAs and DnaKs are the residues that in the SBDβ form an arch over the backbone of the bound peptide. In *E. coli* DnaK the arch is formed by M404 at the tip of L_1,2_ and A429 at the tip of L_3,4_ (Figures 6A,B). A429 is highly conserved in all prokaryotic DnaKs. Position 404 is either methionine or leucine in DnaKs. For *E. coli* DnaK it was shown that replacing either of these residues modulates substrate specificity (Rüdiger et al., 2000). In eukaryotic cytosolic Hsp70s there is alanine in the position that corresponds to 404 in DnaK and tyrosine in position 429. Thus, the small and large hydrophobic residues are reversed in the arch of eukaryotic Hsp70s. In HscAs there is methionine in position 401 corresponding to 404 in DnaK but mostly phenylalanine in position 426 corresponding to 429 of DnaK. Thus, HscAs have large hydrophobic residues in both positions of the arch. The recognition sequence specificity of HscAs seems to be much more restricted than the promiscuous motif of DnaK (Hoff et al., 2002). In fact, the only client known for HscA is IscU the scaffold protein for the assembly of Fe-S-clusters and within IscU only a single segment LPPVK is bound (Figures 6C,D) (Tapley et al., 2006). Replacement of F426 by alanine increases the K_D_ for a peptide that contains the LPPVK motif by some 6-fold. However, the arch residue F426 is not solely responsible for substrate specificity. The substrate binding pocket of HscA is much shallower than the pocket of DnaK because V436 in DnaK is replaced by methionine (M433) in HscA. Replacement of V436 in DnaK by phenylalanine increases the K_D_ for high-affinity substrate peptides by 40-fold, indicating that a larger sidechain in position 436 in DnaK reduces association of peptides with large hydrophobic sidechains into the binding pocket. In the crystal structure of the SBD of HscA in complex with the IscU sequence-derived peptide ELPPVKI P4 was bound in the central pocket. This proline was absolutely essential for binding to HscA and could not be replaced by any other residue (Tapley et al., 2006). Replacement of M433 in HscA with valine reduced the affinity for the ELPPVKI peptide to 1/5th of the affinity of wild-type HscA and increased the affinity for the typical DnaK binding peptide NRLLLTG with a central leucine, thus, this replacement leads to a loss of specificity (Tapley et al., 2006). So, it appears that the shallower hydrophobic pocket is tailored for proline and selects against larger hydrophobic sidechains. The reduced interaction interface between proline and the binding pocket, which does not provide enough binding energy for high-affinity binding, is compensated by the larger phenylalanine in the arch. Other residues lining the binding pocket, like F426 and I438 in DnaK are conserved in HscA (F423 and I435). In DnaK I438, together with V440, L484 and D148 were implicated in the mechanism of substrate stimulation of ATP hydrolysis (Kityk et al., 2015) (Figure 3D). All of these residues are conserved in HscAs and *E. coli* HscA’s ATPase activity was stimulated synergistically by IscU and the HscA-specific JDP HscB (Silberg et al., 2004). The crystal structure of the SBD of HscA in complex with the ELPPVKI peptide also revealed that the peptide bound in the reverse orientation as compared to a peptide bound to DnaK’s SBD (Figures 6C,D) and the prolines in the substrate were responsible for this orientation. In fact, proline containing peptides can also bind in the reverse orientation to DnaK (Zahn et al., 2013). Nevertheless, the peptides are bound in both cases *via* hydrogen bonds to the substrate peptide backbone (Figures 6B,D). An additional structural difference that should increase the intrinsic substrate dissociation rate is the reduced interaction of helix B of the SBDα with the outer loops L_3,4_ and L_5,6_. Whereas in DnaK the latch is formed by four polar interactions between D431 and H544 and R467 and D540, only a single hydrogen bond was found in the crystal structure of the SBD of HscA (Figures 6B,D). Also, a hydrogen bond between N537 and M404 in DnaK is missing in HscAs further weakening the interaction of the lid with the SBDβ.

Another structural difference between DnaKs and HscAs is the C-terminal intrinsically disordered tail that is only seven residues long in most HscAs as compared to some 30 residues in DnaKs. Since these residues may be involved in the interaction with clients, such differences might be significant. In DnaKs the tail is highly charged with on average seven negatively and three positively charged residues in the last 18 residues (Figure 8B). The intrinsic disorder and the charge might allow low-affinity binding to misfolded proteins. Since HscAs are specialized for one or a small number of proteins, they may not need such an additional “tentacle” for interaction.

HscAs seem to be highly specialized to chaperoning the transfer of Fe-S-clusters from IscU to an apo-enzyme in cooperation with the JDP HscB [for review see (Puglisi and Pastore, 2018)]. Whereas HscAs are only found in bacteria, HscB homologs also exist in mitochondria where they either interact with the generalist DnaK-type Hsp70 (in most eukaryotic cells) or with a specialized Hsp70 (some fungi) that emerged by convergent evolution but is still more DnaK-like than HscA-like (Schilke et al., 2006; Kleczewska et al., 2020).

In *E. coli* HscC the GrpE signature is also absent and GrpE interacting residues are not conserved, suggesting the HscCs also do not interact with GrpE (Figure 8C). Consistently, both salt bridges that bridge the nucleotide binding cleft in DnaK are absent in most HscCs and it can be expected that their nucleotide dissociation rate is equally high as in *E. coli* HscA. However, some HscCs seem to have the lower salt bridge like eukaryotic Hsp70s and are therefore expected to have an only 20-fold increased intrinsic nucleotide dissociation rate. In HscCs a second region is deleted in the NBD lobe I corresponding to residue 76–101 in DnaK, resulting in a further opening of the ATP binding cleft. The functional relevance of this deletion is not clear. Interestingly, a similar deletion is found also in many DnaK-type bacterial Hsp70s outside the proteobacterial clade. HscCs have in addition a few smaller deletions in the NBD, one of which affects a loop close to the binding site for J-domains and contains in DnaK a J-domain interacting residue (D211) (Figure 8C). Furthermore, HscCs have an insertion of 4–8 amino acids in L_1,2_ in the SBDβ and a corresponding deletion of nine residues in helix B of the SBDα (Kluck et al., 2002). Such changes should have a significant influence on client binding and specificity. But in contrast to HscA, the sequence specificity seems to be broader in HscCs as peptide library scanning revealed (Kluck et al., 2002), suggesting that HscCs have a more diverse client spectrum. The amino acid preference of *E. coli* HscC in binding peptides is similar to the preference of *E. coli* DnaK, except for leucine which is strongly preferred by DnaK but not enriched in binding peptides for HscC. The by far major contribution to binding to HscC was a positive charge. Finally, the C-terminal disordered tail is with some 10–15 residues slightly longer than the tails of HscAs and contains negative charges and aliphatic residues and therefore could contribute a low-affinity client binding site (Figure 8B).

The ATPase activity of *E. coli* HscC was only stimulated by DjlC and not by DnaJ, CbpA or HscB, suggesting that HscC cooperates with DjlC but not with JDP known to interact with DnaK or HscA. Since DjlB is over the entire sequence 50% identical to DjlC and 64% within the J-domain as compared to 23, 25, 23, and 16% identity to the J-domains of the DnaK cochaperones DnaJ, CbpA, DjlA and the HscA cochaperone HscB, respectively, and since the DjlB and DjlC encoding genes are located in close proximity of the HscC encoding gene on the *E. coli* chromosome, it can be assumed that HscC also cooperates with DjlB. Both, DjlB and DjlC are tail-anchored proteins and for DjlC is was shown that it is inserted into the plasma membrane (Peschke et al., 2018). This suggests a function of HscC at the plasma membrane, possibly with cytosolic domains of transmembrane proteins. Deletion of HscC as well as deletion of both DjlB and DjlC encoding genes result in elevated sensitivity to Cd^2+^ ions. Through studies in yeast it was shown that Cd^2+^ toxicity is due to the induction of aggregation of newly synthesized proteins (Jacobson et al., 2017). These aggregates are most likely solubilized and the proteins refolded by the DnaK, DnaJ, GrpE and the AAA+ protein ClpB (Goloubinoff et al., 1999). Since many of the residues in DnaK that interact with ClpB (Rosenzweig et al., 2013) are not conserved in HscC, it is rather unlikely that HscC participates in solubilization of Cd^2+^-induced protein aggregates. These data suggest that HscC together with DjlB and DjlC are chaperoning proteins, most likely transmembrane proteins, that aid in the detoxification of Cd^2+^ ions by sequestration or export.

It seems rather unlikely that the detoxification of Cd^2+^ ions is the only raison d’être for HscC proteins. It is imaginable that efflux pumps for antibiotics could be other clients of HscC. This has not been tested so far. A closer look at the distribution of HscC proteins in the phylogenetic tree of prokaryotes might also give a hint for their functional importance (Barriot et al., 2020). How a membrane localized function of HscC increases UV resistance is also unclear.

Taken together, HscAs and HscCs have evolved for tasks clearly distinct from the physiological role of DnaKs. For these tasks they coevolved with specialized JDPs as targeting factors. Since HscAs and HscCs have a limited number of clients, their ATPase motor could be tuned to be optimal for the respective tasks and a nucleotide exchange factors was then not anymore necessary. These data suggest that nucleotide exchange factors are important for chaperoning a wide variety of clients that have different structures and folding kinetics.

Neither HscA nor HscC are found in eukaryota, as mentioned above. The most likely reason is that the ancestors of eukaryota did have neither HscA nor HscC. According to a phylogenetic analysis the occurrence of HscA and HscC in the prokaryotic tree of life is quite sporadic (Barriot et al., 2020), suggesting acquisition of the genes by horizontal gene transfer. The spreading of HscA and HscC could have started after the segregation of prokaryota and eukaryota. Such a hypothesis could be substantiated by more extensive sequence analysis.

## Prokaryotic Hsp70s as Drug Targets

The spreading of resistance against multiple antibiotics in many pathogenic bacteria poses a serious threat to public health. New target structures for the development of novel antimicrobial substances are urgently needed. Could Hsp70 be such a new target? Deletion of the genes encoding HscA and HscC in *E. coli* did not affect viability greatly. Targeting them is therefore not expected to result in severe growth inhibition. Though, iron limitation during infection of a multicellular host might make HscA essential. This needs to be tested. In contrast, deletion of *dnaK* results in filamentous growth and temperature sensitivity above 35°C. Although the filamentous growth can be compensated by suppressor mutations in the rpoH gene encoding the heat shock transcription factor σ^32^, this is not expected to alleviate the selection pressure on pathogenic bacteria upon infection of a multicellular host, as this most likely is associated with severe stress conditions that require a fully functional heat shock response for survival. Pathogenic bacteria are even more dependent on DnaK and virulence of many pathogens is particularly sensitive to a loss of DnaK function, as mentioned above. Furthermore, dormancy is a common strategy of bacteria to evade host defense mechanisms and antibiotic treatment, and is responsible for persistent infections. DnaK is not only one of the most important proteins for the formation of the persistence state in the presences of a variety of antibiotics targeting different cellular processes but also for regrowth out of the dormant state (Wu et al., 2015; Pu et al., 2018). Thus, DnaK appears to be a suitable, currently unexplored target for the development of novel antimicrobial drugs. However, Hsp70s are highly conserved in evolution, as mentioned above, and Hsp70s are essential under all conditions in eukaryotic organisms. It is therefore important to explore whether drugs could be developed that distinguish DnaK from eukaryotic Hsp70s as to abrogate growth of the pathogen without jeopardizing protein homeostasis in the eukaryotic host. Despite the high degree of conservation, specific targeting of Hsp70s seems to be possible as a compound was already identified that specifically inhibits human Hsp70 but is much less active against *E. coli* DnaK (Hassan et al., 2015). The reverse may also be possible.

Specific DnaK binding agents were found in the cocktail of antimicrobial peptides that are part of the innate immune system of insects. Apidaecin, drosocin, oncocins and pyrrhocoricin are examples of cell membrane-penetrating proline-rich peptides of 18–20 amino acid that inhibit the growth of *several Gram-negative bacteria* at a minimal inhibitory concentration of 2–8 µM (Scocchi et al., 2011). Similar proline-rich peptides and small proteins were also found in vertebrates. Some of the proline-rich antimicrobial peptides were shown to bind like a substrate peptide to the SBD of DnaK but not to human Hsp70 (Otvos et al., 2000; Zahn et al., 2013). However, whether DnaK is the primary target of these antimicrobial peptides in bacteria is not clear as it was reported that apidaecin and oncocin and derivatives thereof were equally active in a *∆dnaK* strain, suggesting a DnaK-independent mode of action (Czihal et al., 2012; Krizsan et al., 2014).

In-vitro-screening for modulators of the ATPase activity of DnaK identified small molecule activators and inhibitors (Chang et al., 2008b; Wisén et al., 2008; Wisén and Gestwicki, 2008; Wisén et al., 2010). Whether these small molecules are specific for DnaK or whether they also target human Hsp70 is not clear. Efforts for finding Hsp70 modulators focus currently more on the human homologs, since Hsp70 is an important pro-survival factor. Cancer cells seem to be addicted to Hsp70s and inhibition of Hsp70 appears to be a viable strategy to combat tumor growth and survival (Kumar et al., 2016; Gestwicki and Shao, 2018; Albakova et al., 2020). On the other side, activators of Hsp70 might be beneficial in neurodegenerative disorders to counteract protein misfolding and amyloid fibril formation and to promote disassembly of amorphous protein aggregates and amyloid fibrils (Davis et al., 2020). In the light of the current multi-antibiotic resistance crisis more efforts should be devoted to develop inhibitors for the bacterial Hsp70. For Hsp90 it was shown that development of resistance to antimicrobial drugs depends on this chaperone (Cowen and Lindquist, 2005). This seems to be part of the capacity of this chaperone to buffer evolvability and plasticity of organisms. In analogy, it can be expected that DnaK in bacteria serve similar functions, even more so as Hsp90 in bacteria is not essential and absent in many prokaryotic species.

## References

[B1] Aguilar-Rodríguez J. Sabater-Muñoz B. Montagud-Martínez R. Berlanga V. Alvarez-Ponce D. Wagner A. (2016). The Molecular Chaperone DnaK Is a Source of Mutational Robustness. Genome Biol. Evol. 8, 2979–2991. 10.1093/gbe/evw176 27497316 PMC5630943

[B2] Ahmad A. Bhattacharya A. McDonald R. A. Cordes M. Ellington B. Bertelsen E. B. (2011). Heat Shock Protein 70 kDa chaperone/DnaJ Cochaperone Complex Employs an Unusual Dynamic Interface. Proc. Natl. Acad. Sci. 108, 18966–18971. 10.1073/pnas.1111220108 22065753 PMC3223468

[B3] Albakova Z. Armeev G. A. Kanevskiy L. M. Kovalenko E. I. Sapozhnikov A. M. (2020). HSP70 Multi-Functionality in Cancer. Cells 9, 587. 10.3390/cells9030587 PMC7140411 32121660

[B4] Alderson T. R. Kim J. H. Cai K. Frederick R. O. Tonelli M. Markley J. L. (2014). The Specialized Hsp70 (HscA) Interdomain Linker Binds to its Nucleotide-Binding Domain and Stimulates ATP Hydrolysis in BothcisandtransConfigurations. Biochemistry 53, 7148–7159. 10.1021/bi5010552 25372495 PMC4245983

[B5] Angelidis C. E. Lazaridis I. Pagoulatos G. N. (1999). Aggregation of Hsp70 and Hsc70in Vivois Distinct and Ï¿½temperature-dependent and Their Chaperone Function Is Ï¿½directly Related to Non-aggregated Forms. Eur. J. Biochem. 259, 505–512. 10.1046/j.1432-1327.1999.00078.x 9914533

[B6] Barends T. R. M. Brosi R. W. W. Steinmetz A. Scherer A. Hartmann E. Eschenbach J. (2013). Combining Crystallography and EPR: crystal and Solution Structures of the Multidomain Cochaperone DnaJ. Acta Crystallogr. D Biol. Cryst. 69, 1540–1552. 10.1107/s0907444913010640 23897477 PMC3727329

[B7] Barriot R. Latour J. Castanié-Cornet M.-P. Fichant G. Genevaux P. (2020). J-domain Proteins in Bacteria and Their Viruses. J. Mol. Biol. 432, 3771–3789. 10.1016/j.jmb.2020.04.014 32305462

[B8] Barthel T. K. Zhang J. Walker G. C. (2001). ATPase-Defective Derivatives of Escherichia coliDnaK that Behave Differently with Respect to ATP-Induced Conformational Change and Peptide Release. J. Bacteriol. 183, 5482–5490. 10.1128/jb.183.19.5482-5490.2001 11544208 PMC95437

[B9] Benaroudj N. Batelier G. Triniolles F. Ladjimi M. M. (1995). Self-Association of the Molecular Chaperone HSC70. Biochemistry 34, 15282–15290. 10.1021/bi00046a037 7578144

[B10] Bertelsen E. B. Chang L. Gestwicki J. E. Zuiderweg E. R. P. (2009). Solution Conformation of Wild-type *E. coli* Hsp70 (DnaK) Chaperone Complexed with ADP and Substrate. Proc. Natl. Acad. Sci. 106, 8471–8476. 10.1073/pnas.0903503106 19439666 PMC2689011

[B11] Bhattacharya K. Weidenauer L. Luengo T. M. Pieters E. C. Echeverria P. C. Bernasconi L. (2020). The Hsp70-Hsp90 Co-chaperone Hop/Stip1 Shifts the Proteostatic Balance from Folding towards Degradation. Nat. Commun. 11, 5975. 10.1038/s41467-020-19783-w 33239621 PMC7688965

[B12] Bienert S. Waterhouse A. de Beer T. A. P. Tauriello G. Studer G. Bordoli L. (2017). The SWISS-MODEL Repository-New Features and Functionality. Nucleic Acids Res. 45, D313–D319. 10.1093/nar/gkw1132 27899672 PMC5210589

[B13] Blond-Elguindi S. Fourie A. M. Sambrook J. F. Gething M. J. (1993). Peptide-dependent Stimulation of the ATPase Activity of the Molecular Chaperone BiP Is the Result of Conversion of Oligomers to Active Monomers. J. Biol. Chem. 268, 12730–12735. 10.1016/s0021-9258(18)31449-2 8509407

[B14] Bracher A. Bracher A. Verghese J. Verghese J. (2015). The Nucleotide Exchange Factors of Hsp70 Molecular Chaperones. Front. Mol. Biosciences 2, 10. 10.3389/fmolb.2015.00010 PMC4753570 26913285

[B15] Brehmer D. Gässler C. Rist W. Mayer M. P. Bukau B. (2004). Influence of GrpE on DnaK-Substrate Interactions. J. Biol. Chem. 279, 27957–27964. 10.1074/jbc.m403558200 15102842

[B16] Brehmer D. Rüdiger S. Gässler C. S. Klostermeier D. Packschies L. Reinstein J. (2001). Tuning of Chaperone Activity of Hsp70 Proteins by Modulation of Nucleotide Exchange. Nat. Struct. Biol. 8, 427–432. 10.1038/87588 11323718

[B17] Bukau B. Walker G. C. (1990). Mutations Altering Heat Shock Specific Subunit of RNA Polymerase Suppress Major Cellular Defects of *E. coli* Mutants Lacking the DnaK Chaperone. EMBO J. 9, 4027–4036. 10.1002/j.1460-2075.1990.tb07624.x 2249663 PMC552175

[B18] Burkholder W. F. Panagiotidis C. A. Silverstein S. J. Cegielska A. Gottesman M. E. Gaitanaris G. A. (1994). Isolation and Characterization of an *Escherichia coli* DnaK Mutant with Impaired ATPase Activity. J. Mol. Biol. 242, 364–377. 10.1006/jmbi.1994.1587 7932696

[B19] Calloni G. Chen T. Schermann S. M. Chang H.-C. Genevaux P. Agostini F. (2012). DnaK Functions as a Central Hub in the *E. coli* Chaperone Network. Cel Rep. 1, 251–264. 10.1016/j.celrep.2011.12.007 22832197

[B20] Chae C. Sharma S. Hoskins J. R. Wickner S. (2004). CbpA, a DnaJ Homolog, Is a DnaK Co-chaperone, and its Activity Is Modulated by CbpM. J. Biol. Chem. 279, 33147–33153. 10.1074/jbc.m404862200 15184371

[B21] Chang L. Bertelsen E. B. Wisén S. Larsen E. M. Zuiderweg E. R. P. Gestwicki J. E. (2008). High-throughput Screen for Small Molecules that Modulate the ATPase Activity of the Molecular Chaperone DnaK. Anal. Biochem. 372, 167–176. 10.1016/j.ab.2007.08.020 17904512

[B22] Chang Y.-W. Sun Y.-J. Wang C. Hsiao C.-D. (2008). Crystal Structures of the 70-kDa Heat Shock Proteins in Domain Disjoining Conformation. J. Biol. Chem. 283, 15502–15511. 10.1074/jbc.m708992200 18400763 PMC3258884

[B23] Chenoweth M. R. Trun N. Wickner S. (2007). *In Vivo* modulation of a DnaJ Homolog, CbpA, by CbpM. Jb 189, 3635–3638. 10.1128/jb.01757-06 PMC1855902 17337578

[B24] Chevalier M. Rhee H. Elguindi E. C. Blond S. Y. (2000). Interaction of Murine BiP/GRP78 with the DnaJ Homologue MTJ1. J. Biol. Chem. 275, 19620–19627. 10.1074/jbc.m001333200 10777498 PMC1534116

[B25] Clerico E. M. Tilitsky J. M. Meng W. Gierasch L. M. (2015). How Hsp70 Molecular Machines Interact with Their Substrates to Mediate Diverse Physiological Functions. J. Mol. Biol. 427, 1575–1588. 10.1016/j.jmb.2015.02.004 25683596 PMC4440321

[B26] Collet C. Thomassin J. L. Francetic O. Genevaux P. Tran Van Nhieu G. (2018). Protein Polarization Driven by Nucleoid Exclusion of DnaK(HSP70)-Substrate Complexes. Nat. Commun. 9, 2027. 10.1038/s41467-018-04414-2 29795186 PMC5966378

[B27] Cowen L. E. Lindquist S. (2005). Hsp90 Potentiates the Rapid Evolution of New Traits: Drug Resistance in Diverse Fungi. Science 309, 2185–2189. 10.1126/science.1118370 16195452

[B28] Crooks G. E. Hon G. Chandonia J. M. Brenner S. E. (2004). WebLogo: a Sequence Logo Generator. Genome Res. 14, 1188–1190. 10.1101/gr.849004 15173120 PMC419797

[B29] Cupp-Vickery J. R. Peterson J. C. Ta D. T. Vickery L. E. (2004). Crystal Structure of the Molecular Chaperone HscA Substrate Binding Domain Complexed with the IscU Recognition Peptide ELPPVKIHC. J. Mol. Biol. 342, 1265–1278. 10.1016/j.jmb.2004.07.025 15351650

[B30] Cupp-Vickery J. R. Vickery L. E. (2000). Crystal Structure of Hsc20, a J-type Co-chaperone from *Escherichia coli* . J. Mol. Biol. 304, 835–845. 10.1006/jmbi.2000.4252 11124030

[B31] Czihal P. Knappe D. Fritsche S. Zahn M. Berthold N. Piantavigna S. (2012). Api88 Is a Novel Antibacterial Designer Peptide to Treat Systemic Infections with Multidrug-Resistant Gram-Negative Pathogens. ACS Chem. Biol. 7, 1281–1291. 10.1021/cb300063v 22594381

[B32] Davis A. K. Pratt W. B. Lieberman A. P. Osawa Y. (2020). Targeting Hsp70 Facilitated Protein Quality Control for Treatment of Polyglutamine Diseases. Cell. Mol. Life Sci. 77, 977–996. 10.1007/s00018-019-03302-2 31552448 PMC7137528

[B33] De Los Rios P. Barducci A. (2014). Hsp70 Chaperones Are Non-equilibrium Machines that Achieve Ultra-affinity by Energy Consumption. eLife 3, e02218. 10.7554/elife.02218 24867638 PMC4030575

[B34] De Los Rios P. Ben-Zvi A. Slutsky O. Azem A. Goloubinoff P. (2006). Hsp70 Chaperones Accelerate Protein Translocation and the Unfolding of Stable Protein Aggregates by Entropic Pulling. Proc. Natl. Acad. Sci. 103, 6166–6171. 10.1073/pnas.0510496103 16606842 PMC1458849

[B35] Deuerling E. Schulze-Specking A. Tomoyasu T. Mogk A. Bukau B. (1999). Trigger Factor and DnaK Cooperate in Folding of Newly Synthesized Proteins. Nature 400, 693–696. 10.1038/23301 10458167

[B36] Döring K. Ahmed N. Riemer T. Suresh H. G. Vainshtein Y. Habich M. (2017). Profiling Ssb-Nascent Chain Interactions Reveals Principles of Hsp70-Assisted Folding. Cell 170, 298–311.e20. 10.1016/j.cell.2017.06.038 28708998 PMC7343536

[B37] Elefant F. Palter K. B. (1999). Tissue-specific Expression of Dominant Negative MutantDrosophila HSC70 Causes Developmental Defects and Lethality. MBoC 10, 2101–2117. 10.1091/mbc.10.7.2101 10397752 PMC25422

[B38] English C. A. Sherman W. Meng W. Gierasch L. M. (2017). The Hsp70 Interdomain Linker Is a Dynamic Switch that Enables Allosteric Communication between Two Structured Domains. J. Biol. Chem. 292, 14765–14774. 10.1074/jbc.m117.789313 28754691 PMC5592658

[B39] Evans M. L. Schmidt J. C. Ilbert M. Doyle S. M. Quan S. Bardwell J. C. A. (2011). *E. coli* Chaperones DnaK, Hsp33 and Spy Inhibit Bacterial Functional Amyloid Assembly. Prion 5, 323–334. 10.4161/pri.5.4.18555 22156728 PMC3821533

[B40] Faust O. Abayev-Avraham M. Wentink A. S. Maurer M. Nillegoda N. B. London N. (2020). HSP40 Proteins Use Class-specific Regulation to Drive HSP70 Functional Diversity. Nature 587, 489–494. 10.1038/s41586-020-2906-4 33177718

[B41] Fauvet B. Finka A. Castanie-Cornet M. P. Cirinesi A. M. Genevaux P. Quadroni M. (2021). Bacterial Hsp90 Facilitates the Degradation of Aggregation-Prone Hsp70-Hsp40 Substrates. Front. Mol. Biosci. 8, 653073. 10.3389/fmolb.2021.653073 33937334 PMC8082187

[B42] Flaherty K. M. DeLuca-Flaherty C. McKay D. B. (1990). Three-dimensional Structure of the ATPase Fragment of a 70K Heat-Shock Cognate Protein. Nature 346, 623–628. 10.1038/346623a0 2143562

[B43] Freiden P. J. Gaut J. R. Hendershot L. M. (1992). Interconversion of Three Differentially Modified and Assembled Forms of BiP. EMBO J. 11, 63–70. 10.1002/j.1460-2075.1992.tb05028.x 1740116 PMC556426

[B44] Gao X. Carroni M. Nussbaum-Krammer C. Mogk A. Nillegoda N. B. Szlachcic A. (2015). Human Hsp70 Disaggregase Reverses Parkinson's-Linked α-Synuclein Amyloid Fibrils. Mol. Cel 59, 781–793. 10.1016/j.molcel.2015.07.012 PMC5072489 26300264

[B45] Genest O. Hoskins J. R. Camberg J. L. Doyle S. M. Wickner S. (2011). Heat Shock Protein 90 from *Escherichia coli* Collaborates with the DnaK Chaperone System in Client Protein Remodeling. Proc. Natl. Acad. Sci. 108, 8206–8211. 10.1073/pnas.1104703108 21525416 PMC3100916

[B46] Gestwicki J. E. Shao H. (2018). Inhibitors and Chemical Probes for Molecular Chaperone Networks. J. Biol. Chem. 294, 2151–2161. 10.1074/jbc.TM118.002813 30213856 PMC6369302

[B47] Ghazaei C. (2017). Role and Mechanism of the Hsp70 Molecular Chaperone Machines in Bacterial Pathogens. J. Med. Microbiol. 66, 259–265. 10.1099/jmm.0.000429 28086078

[B48] Goloubinoff P. Mogk A. Zvi A. P. B. Tomoyasu T. Bukau B. (1999). Sequential Mechanism of Solubilization and Refolding of Stable Protein Aggregates by a Bichaperone Network. Proc. Natl. Acad. Sci. 96, 13732–13737. 10.1073/pnas.96.24.13732 10570141 PMC24133

[B49] Goloubinoff P. Sassi A. S. Fauvet B. Barducci A. De Los Rios P. (2018). Chaperones Convert the Energy from ATP into the Nonequilibrium Stabilization of Native Proteins. Nat. Chem. Biol. 14, 388–395. 10.1038/s41589-018-0013-8 29507388

[B50] Gowda N. K. C. Kaimal J. M. Kityk R. Daniel C. Liebau J. Öhman M. (2018). Nucleotide Exchange Factors Fes1 and HspBP1 Mimic Substrate to Release Misfolded Proteins from Hsp70. Nat. Struct. Mol. Biol. 25, 83–89. 10.1038/s41594-017-0008-2 29323280

[B51] Grimshaw J. P. A. Jelesarov I. Schönfeld H.-J. Christen P. (2001). Reversible thermal Transition in GrpE, the Nucleotide Exchange Factor of the DnaK Heat-Shock System. J. Biol. Chem. 276, 6098–6104. 10.1074/jbc.m009290200 11084044

[B52] Grimshaw J. P. A. Jelesarov I. Siegenthaler R. K. Christen P. (2003). Thermosensor Action of GrpE. J. Biol. Chem. 278, 19048–19053. 10.1074/jbc.m300924200 12639955

[B53] Groemping Y. Reinstein J. (2001). Folding Properties of the Nucleotide Exchange Factor GrpE from Thermus Thermophilus: GrpE Is a Thermosensor that Mediates Heat Shock Response. J. Mol. Biol. 314, 167–178. 10.1006/jmbi.2001.5116 11724541

[B54] Hanawa T. Yamanishi S. Murayama S. Yamamoto T. Kamiya S. (2002). Participation of DnaK in Expression of Genes Involved in Virulence ofListeria Monocytogenes. FEMS Microbiol. Lett. 214, 69–75. 10.1111/j.1574-6968.2002.tb11326.x 12204374

[B55] Harrison C. J. Hayer-Hartl M. Di Liberto M. Hartl F. Kuriyan J. (1997). Crystal Structure of the Nucleotide Exchange Factor GrpE Bound to the ATPase Domain of the Molecular Chaperone DnaK. Science 276, 431–435. 10.1126/science.276.5311.431 9103205

[B56] Hassan A. Q. Kirby C. A. Zhou W. Schuhmann T. Kityk R. Kipp D. R. (2015). The Novolactone Natural Product Disrupts the Allosteric Regulation of Hsp70. Chem. Biol. 22, 87–97. 10.1016/j.chembiol.2014.11.007 25544045

[B57] Hesterkamp T. Bukau B. (1998). Role of the DnaK and HscA Homologs of Hsp70 Chaperones in Protein Folding in E.Coli. EMBO J. 17, 4818–4828. 10.1093/emboj/17.16.4818 9707441 PMC1170811

[B58] Hoff K. G. Ta D. T. Tapley T. L. Silberg J. J. Vickery L. E. (2002). Hsc66 Substrate Specificity Is Directed toward a Discrete Region of the Iron-Sulfur Cluster Template Protein IscU. J. Biol. Chem. 277, 27353–27359. 10.1074/jbc.m202814200 11994302

[B59] Imamoglu R. Balchin D. Hayer-Hartl M. Hartl F. U. (2020). Bacterial Hsp70 Resolves Misfolded States and Accelerates Productive Folding of a Multi-Domain Protein. Nat. Commun. 11, 365. 10.1038/s41467-019-14245-4 31953415 PMC6969021

[B60] Ishiai M. Wada C. Kawasaki Y. Yura T. (1994). Replication Initiator Protein RepE of Mini-F Plasmid: Functional Differentiation between Monomers (Initiator) and Dimers (Autogenous Repressor). Proc. Natl. Acad. Sci. 91, 3839–3843. 10.1073/pnas.91.9.3839 8170998 PMC43677

[B61] Jacobson T. Priya S. Sharma S. K. Andersson S. Jakobsson S. Tanghe R. (2017). Cadmium Causes Misfolding and Aggregation of Cytosolic Proteins in Yeast. Mol. Cel Biol 37. 10.1128/mcb.00490-16 PMC5559669 28606932

[B62] Jha J. K. Li M. Ghirlando R. Miller Jenkins L. M. Wlodawer A. Chattoraj D. (2017). The DnaK Chaperone Uses Different Mechanisms to Promote and Inhibit Replication of *Vibrio cholerae* Chromosome 2. mBio 8. 10.1128/mbio.00427-17 PMC5395669 28420739

[B63] Jiang Y. Rossi P. Kalodimos C. G. (2019). Structural Basis for Client Recognition and Activity of Hsp40 Chaperones. Science 365, 1313–1319. 10.1126/science.aax1280 31604242 PMC7023980

[B64] Kadibalban A. S. Bogumil D. Landan G. Dagan T. (2016). DnaK-Dependent Accelerated Evolutionary Rate in Prokaryotes. Genome Biol. Evol. 8, 1590–1599. 10.1093/gbe/evw102 27189986 PMC4898814

[B65] Kampinga H. H. Craig E. A. (2010). The HSP70 Chaperone Machinery: J Proteins as Drivers of Functional Specificity. Nat. Rev. Mol. Cel Biol 11, 579–592. 10.1038/nrm2941 PMC3003299 20651708

[B66] Kelley W. L. Georgopoulos C. (1997). The T/t Common Exon of Simian Virus 40, JC, and BK Polyomavirus T Antigens Can Functionally Replace the J-Domain of the *Escherichia coli* DnaJ Molecular Chaperone. Proc. Natl. Acad. Sci. 94, 3679–3684. 10.1073/pnas.94.8.3679 9108037 PMC20500

[B67] Kellner R. Hofmann H. Barducci A. Wunderlich B. Nettels D. Schuler B. (2014). Single-molecule Spectroscopy Reveals Chaperone-Mediated Expansion of Substrate Protein. Proc. Natl. Acad. Sci. USA 111, 13355–13360. 10.1073/pnas.1407086111 25165400 PMC4169939

[B68] King C. Eisenberg E. Greene L. (1995). Polymerization of 70-kDa Heat Shock Protein by Yeast DnaJ in ATP. J. Biol. Chem. 270, 22535–22540. 10.1074/jbc.270.38.22535 7673245

[B69] Kityk R. Kopp J. Mayer M. P. (2018). Molecular Mechanism of J-Domain-Triggered ATP Hydrolysis by Hsp70 Chaperones. Mol. Cel 69, 227–237.e4. 10.1016/j.molcel.2017.12.003 29290615

[B70] Kityk R. Kopp J. Sinning I. Mayer M. P. (2012). Structure and Dynamics of the ATP-Bound Open Conformation of Hsp70 Chaperones. Mol. Cel 48, 863–874. 10.1016/j.molcel.2012.09.023 23123194

[B71] Kityk R. Vogel M. Schlecht R. Bukau B. Mayer M. P. (2015). Pathways of Allosteric Regulation in Hsp70 Chaperones. Nat. Commun. 6, 8308. 10.1038/ncomms9308 26383706 PMC4595643

[B72] Kleczewska M. Grabinska A. Jelen M. Stolarska M. Schilke B. Marszalek J. (2020). Biochemical Convergence of Mitochondrial Hsp70 System Specialized in Iron-Sulfur Cluster Biogenesis. Int. J. Mol. Sci. 21. 10.3390/ijms21093326 PMC7247549 32397253

[B73] Kluck C. J. Patzelt H. Genevaux P. Brehmer D. Rist W. Schneider-Mergener J. (2002). Structure-Function Analysis of HscC, theEscherichia Coli Member of a Novel Subfamily of Specialized Hsp70 Chaperones. J. Biol. Chem. 277, 41060–41069. 10.1074/jbc.m206520200 12183460

[B74] Kmiecik S. W. Le Breton L. Mayer M. P. (2020). Feedback Regulation of Heat Shock Factor 1 (Hsf1) Activity by Hsp70-Mediated Trimer Unzipping and Dissociation from DNA. EMBO J. 39, e104096. 10.15252/embj.2019104096 32490574 PMC7360973

[B75] Kobayashi Y. Ohtsu I. Fujimura M. Fukumori F. (2011). A Mutation in dnaK Causes Stabilization of the Heat Shock Sigma Factor σ32, Accumulation of Heat Shock Proteins and Increase in Toluene-Resistance in Pseudomonas Putida. Environ. Microbiol. 13, 2007–2017. 10.1111/j.1462-2920.2010.02344.x 20880327

[B76] Köhler S. Teyssier J. Cloeckaert A. Rouot B. Liautard J.-P. (1996). Participation of the Molecular Chaperone DnaK in Intracellular Growth of Brucella Suis within U937-Derived Phagocytes. Mol. Microbiol. 20, 701–712. 10.1111/j.1365-2958.1996.tb02510.x 8793869

[B77] Krizsan A. Volke D. Weinert S. Sträter N. Knappe D. Hoffmann R. (2014). Insect-Derived Proline-Rich Antimicrobial Peptides Kill Bacteria by Inhibiting Bacterial Protein Translation at the 70 S Ribosome. Angew. Chem. Int. Ed. 53, 12236–12239. 10.1002/anie.201407145 25220491

[B78] Kumar A. Tiwari A. K. (2018). Molecular Chaperone Hsp70 and its Constitutively Active Form Hsc70 Play an Indispensable Role during Eye Development of *Drosophila melanogaster* , Mol. Neurobiol. 55(5):4345–4361. 10.1007/s12035-017-0650-z 28634860

[B79] Kumar D. P. Vorvis C. Sarbeng E. B. Cabra Ledesma V. C. Willis J. E. Liu Q. (2011). The Four Hydrophobic Residues on the Hsp70 Inter-domain Linker Have Two Distinct Roles. J. Mol. Biol. 411, 1099–1113. 10.1016/j.jmb.2011.07.001 21762702 PMC5737734

[B80] Kumar M. Sourjik V. (2012). Physical Map and Dynamics of the Chaperone Network in *Escherichia coli* . Mol. Microbiol. 84, 736–747. 10.1111/j.1365-2958.2012.08054.x 22463727

[B81] Kumar S. Stokes J. Singh U. P. Scissum Gunn K. Acharya A. Manne U. (2016). Targeting Hsp70: A Possible Therapy for Cancer. Cancer Lett. 374, 156–166. 10.1016/j.canlet.2016.01.056 26898980 PMC5553548

[B82] Lagaudrière-Gesbert C. Newmyer S. L. Gregers T. F. Bakke O. Ploegh H. L. (2002). Uncoating ATPase Hsc70 Is Recruited by Invariant Chain and Controls the Size of Endocytic Compartments. Proc. Natl. Acad. Sci. 99, 1515–1520. 10.1073/pnas.042688099 11818572 PMC122222

[B83] Lai A. L. Clerico E. M. Blackburn M. E. Patel N. A. Robinson C. V. Borbat P. P. (2017). Key Features of an Hsp70 Chaperone Allosteric Landscape Revealed by Ion-Mobility Native Mass Spectrometry and Double Electron-Electron Resonance. J. Biol. Chem. 292, 8773–8785. 10.1074/jbc.m116.770404 28428246 PMC5448104

[B84] Langer T. Lu C. Echols H. Flanagan J. Hayer M. K. Hartl F. U. (1992). Successive Action of DnaK, DnaJ and GroEL along the Pathway of Chaperone-Mediated Protein Folding. Nature 356, 683–689. 10.1038/356683a0 1349157

[B85] Laufen T. Mayer M. P. Beisel C. Klostermeier D. Mogk A. Reinstein J. (1999). Mechanism of Regulation of Hsp70 Chaperones by DnaJ Cochaperones. Proc. Natl. Acad. Sci. 96, 5452–5457. 10.1073/pnas.96.10.5452 10318904 PMC21880

[B86] Li J. Qian X. Sha B. (2003). The crystal Structure of the Yeast Hsp40 Ydj1 Complexed with its Peptide Substrate. Structure 11, 1475–1483. 10.1016/j.str.2003.10.012 14656432

[B87] Li J. Wu Y. Qian X. Sha B. (2006). Crystal Structure of Yeast Sis1 Peptide-Binding Fragment and Hsp70 Ssa1 C-Terminal Complex. Biochem. J. 398, 353–360. 10.1042/bj20060618 16737444 PMC1559466

[B88] Liberek K. Wall D. Georgopoulos C. (1995). The DnaJ Chaperone Catalytically Activates the DnaK Chaperone to Preferentially Bind the Sigma 32 Heat Shock Transcriptional Regulator. Proc. Natl. Acad. Sci. 92, 6224–6228. 10.1073/pnas.92.14.6224 7603976 PMC41490

[B89] Linke K. Wolfram T. Bussemer J. Jakob U. (2003). The Roles of the Two Zinc Binding Sites in DnaJ. J. Biol. Chem. 278, 44457–44466. 10.1074/jbc.m307491200 12941935

[B90] Liu B. Han Y. Qian S.-B. (2013). Cotranslational Response to Proteotoxic Stress by Elongation Pausing of Ribosomes. Mol. Cel 49, 453–463. 10.1016/j.molcel.2012.12.001 PMC3570626 23290916

[B91] Liu J.-S. Kuo S.-R. Makhov A. M. Cyr D. M. Griffith J. D. Broker T. R. (1998). Human Hsp70 and Hsp40 Chaperone Proteins Facilitate Human Papillomavirus-11 E1 Protein Binding to the Origin and Stimulate Cell-free DNA Replication. J. Biol. Chem. 273, 30704–30712. 10.1074/jbc.273.46.30704 9804845

[B92] Maillot N. J. Honoré F. A. Byrne D. Méjean V. Genest O. (2019). Cold Adaptation in the Environmental Bacterium Shewanella Oneidensis Is Controlled by a J-Domain Co-chaperone Protein Network. Commun. Biol. 2, 323. 10.1038/s42003-019-0567-3 31482142 PMC6715715

[B93] Maisnier-Patin S. Roth J. R. Fredriksson A. Nyström T. Berg O. G. Andersson D. I. (2005). Genomic Buffering Mitigates the Effects of Deleterious Mutations in Bacteria. Nat. Genet. 37, 1376–1379. 10.1038/ng1676 16273106

[B94] Malinverni D. Jost Lopez A. De Los Rios P. Hummer G. Barducci A. (2017). Modeling Hsp70/Hsp40 Interaction by Multi-Scale Molecular Simulations and Co-evolutionary Sequence Analysis. eLife 6, e23471. 10.7554/elife.23471 28498104 PMC5519331

[B95] Malinverni D. Marsili S. Barducci A. De Los Rios P. (2015). Large-Scale Conformational Transitions and Dimerization Are Encoded in the Amino-Acid Sequences of Hsp70 Chaperones. PLoS Comput. Biol. 11, e1004262. 10.1371/journal.pcbi.1004262 26046683 PMC4457872

[B96] Marcinowski M. Höller M. Feige M. J. Baerend D. Lamb D. C. Buchner J. (2011). Substrate Discrimination of the Chaperone BiP by Autonomous and Cochaperone-Regulated Conformational Transitions. Nat. Struct. Mol. Biol. 18, 150–158. 10.1038/nsmb.1970 21217698

[B97] Mashaghi A. Bezrukavnikov S. Minde D. P. Wentink A. S. Kityk R. Zachmann-Brand B. (2016). Alternative Modes of Client Binding Enable Functional Plasticity of Hsp70. Nature 539, 448–451. 10.1038/nature20137 27783598

[B98] Matsui M. Takaya A. Yamamoto T. (2008). σ32-Mediated Negative Regulation of Salmonella Pathogenicity Island 1 Expression. Jb 190, 6636–6645. 10.1128/jb.00744-08 PMC2566199 18723621

[B99] Mayer M. P. Schröder H. Rüdiger S. Paal K. Laufen T. Bukau B. (2000). Multistep Mechanism of Substrate Binding Determines Chaperone Activity of Hsp70. Nat. Struct. Biol. 7, 586–593. 10.1038/76819 10876246

[B100] Mayer M. P. Gierasch L. M. (2019). Recent Advances in the Structural and Mechanistic Aspects of Hsp70 Molecular Chaperones. J. Biol. Chem. 294, 2085–2097. 10.1074/jbc.rev118.002810 30455352 PMC6369304

[B101] Mayer M. P. (2018). Intra-molecular Pathways of Allosteric Control in Hsp70s. Phil. Trans. R. Soc. B 373, 20170183–20170188. 10.1098/rstb.2017.0183 29735737 PMC5941178

[B102] Mayer M. P. Laufen T. Paal K. McCarty J. S. Bukau B. (1999). Investigation of the Interaction between DnaK and DnaJ by Surface Plasmon Resonance Spectroscopy. J. Mol. Biol. 289, 1131–1144. 10.1006/jmbi.1999.2844 10369787

[B103] Mayhew M. da Silva A. C. R. Martin J. Erdjument-Bromage H. Tempst P. Hartl F. U. (1996). Protein Folding in the central Cavity of the GroEL-GroES Chaperonin Complex. Nature 379, 420–426. 10.1038/379420a0 8559246

[B104] McCarty J. S. Buchberger A. Reinstein J. Bukau B. (1995). The Role of ATP in the Functional Cycle of the DnaK Chaperone System. J. Mol. Biol. 249, 126–137. 10.1006/jmbi.1995.0284 7776367

[B105] Meimaridou E. Gooljar S. B. Chapple J. P. (2009). From Hatching to Dispatching: the Multiple Cellular Roles of the Hsp70 Molecular Chaperone Machinery. J. Mol. Endocrinol. 42, 1–9. 10.1677/jme-08-0116 18852216

[B106] Mendoza J. A. Rogers E. Lorimer G. H. Horowitz P. M. (1991). Chaperonins Facilitate the *In Vitro* Folding of Monomeric Mitochondrial Rhodanese. J. Biol. Chem. 266, 13044–13049. 10.1016/s0021-9258(18)98800-9 1677004

[B107] Mogk A. Tomoyasu T. Goloubinoff P. Rüdiger S. Röder D. Langen H. (1999). Identification of Thermolabile *Escherichia coli* Proteins: Prevention and Reversion of Aggregation by DnaK and ClpB. EMBO J. 18, 6934–6949. 10.1093/emboj/18.24.6934 10601016 PMC1171757

[B108] Mokranjac D. Sichting M. Neupert W. Hell K. (2003). Tim14, a Novel Key Component of the Import Motor of the TIM23 Protein Translocase of Mitochondria. EMBO J. 22, 4945–4956. 10.1093/emboj/cdg485 14517234 PMC204468

[B109] Montgomery D. L. Morimoto R. I. Gierasch L. M. (1999). Mutations in the Substrate Binding Domain of the *Escherichia coli* 70 Kda Molecular Chaperone, DnaK, Which Alter Substrate Affinity or Interdomain Coupling 1 1Edited by M. Gottesman. J. Mol. Biol. 286, 915–932. 10.1006/jmbi.1998.2514 10024459

[B110] Morán Luengo T. Kityk R. Mayer M. P. Rüdiger S. G. D. (2018). Hsp90 Breaks the Deadlock of the Hsp70 Chaperone System. Mol. Cel 70, 545–552.e9. 10.1016/j.molcel.2018.03.028 29706537

[B111] Morgan J. R. Prasad K. Jin S. Augustine G. J. Lafer E. M. (2001). Uncoating of Clathrin-Coated Vesicles in Presynaptic Terminals. Neuron 32, 289–300. 10.1016/s0896-6273(01)00467-6 11683998

[B112] Morgner N. Schmidt C. Beilsten-Edmands V. Ebong I.-o. Patel N. A. Clerico E. M. (2015). Hsp70 Forms Antiparallel Dimers Stabilized by Post-translational Modifications to Position Clients for Transfer to Hsp90. Cel Rep. 11, 759–769. 10.1016/j.celrep.2015.03.063 PMC4431665 25921532

[B113] Moro F. Fernández-Sáiz V. Muga A. (2004). The Lid Subdomain of DnaK Is Required for the Stabilization of the Substrate-Binding Site. J. Biol. Chem. 279, 19600–19606. 10.1074/jbc.m400921200 14985342

[B114] Nicoll W. S. Botha M. McNamara C. Schlange M. Pesce E.-R. Boshoff A. (2007). Cytosolic and ER J-Domains of Mammalian and Parasitic Origin Can Functionally Interact with DnaK. Int. J. Biochem. Cel Biol. 39, 736–751. 10.1016/j.biocel.2006.11.006 PMC1906734 17239655

[B115] Nillegoda N. B. Kirstein J. Szlachcic A. Berynskyy M. Stank A. Stengel F. (2015). Crucial HSP70 Co-chaperone Complex Unlocks Metazoan Protein Disaggregation. Nature 524, 247–251. 10.1038/nature14884 26245380 PMC4830470

[B116] Nillegoda N. B. Stank A. Malinverni D. Alberts N. Szlachcic A. Barducci A. (2017). Evolution of an Intricate J-Protein Network Driving Protein Disaggregation in Eukaryotes. eLife 6, e24560. 10.7554/elife.24560 28504929 PMC5542770

[B117] Okuda J. Yamane S. Nagata S. Kunikata C. Suezawa C. Yasuda M. (2017). The *Pseudomonas aeruginosa* dnaK Gene Is Involved in Bacterial Translocation across the Intestinal Epithelial Cell Barrier. Microbiology (Reading) 163, 1208–1216. 10.1099/mic.0.000508 28758636

[B118] Otvos L. Rogers I. O. M. E. Consolvo P. J. Condie B. A. Lovas S. (2000). Interaction between Heat Shock Proteins and Antimicrobial Peptides†. Biochemistry 39, 14150–14159. 10.1021/bi0012843 11087363

[B119] Paek K. H. Walker G. C. (1987). *Escherichia coli* dnaK Null Mutants Are Inviable at High Temperature. J. Bacteriol. 169, 283–290. 10.1128/jb.169.1.283-290.1987 3025174 PMC211765

[B120] Pellecchia M. Szyperski T. Wall D. Georgopoulos C. Wüthrich K. (1996). NMR Structure of the J-Domain and the Gly/Phe-Rich Region of theEscherichia coliDnaJ Chaperone. J. Mol. Biol. 260, 236–250. 10.1006/jmbi.1996.0395 8764403

[B121] Peschke M. Le Goff M. Koningstein G. M. Karyolaimos A. de Gier J.-W. van Ulsen P. (2018). SRP, FtsY, DnaK and YidC Are Required for the Biogenesis of the *E. coli* Tail-Anchored Membrane Proteins DjlC and Flk. J. Mol. Biol. 430, 389–403. 10.1016/j.jmb.2017.12.004 29246766

[B122] Preissler S. Chambers J. E. Crespillo-Casado A. Avezov E. Miranda E. Perez J. (2015). Physiological Modulation of BiP Activity by Trans-protomer Engagement of the Interdomain Linker. eLife 4, e08961. 10.7554/elife.08961 26473973 PMC4608358

[B123] Preissler S. Rato C. Yan Y. Perera L. A. Czako A. Ron D. (2020). Calcium Depletion Challenges Endoplasmic Reticulum Proteostasis by Destabilising BiP-Substrate Complexes. Elife 9. 10.7554/elife.62601 PMC7758071 33295873

[B124] Pu Y. Li Y. Jin X. Tian T. Ma Q. Zhao Z. (2018). ATP-dependent Dynamic Protein Aggregation Regulates Bacterial Dormancy Depth Critical for Antibiotic Tolerance. Mol. Cel 73 (1), 143–156. e4. 10.1016/j.molcel.2018.10.022 30472191

[B125] Puglisi R. Pastore A. (2018). The Role of Chaperones in Iron-Sulfur Cluster Biogenesis. FEBS Lett. 592, 4011–4019. 10.1002/1873-3468.13245 30194723 PMC6506825

[B126] Qi R. Sarbeng E. B. Liu Q. Le K. Q. Xu X. Xu H. (2013). Allosteric Opening of the Polypeptide-Binding Site when an Hsp70 Binds ATP. Nat. Struct. Mol. Biol. 20, 900–907. 10.1038/nsmb.2583 23708608 PMC3772632

[B127] Queitsch C. Sangster T. A. Lindquist S. (2002). Hsp90 as a Capacitor of Phenotypic Variation. Nature 417, 618–624. 10.1038/nature749 12050657

[B128] Rodriguez F. Arsène-Ploetze F. Rist W. Rüdiger S. Schneider-Mergener J. Mayer M. P. (2008). Molecular Basis for Regulation of the Heat Shock Transcription Factor σ32 by the DnaK and DnaJ Chaperones. Mol. Cel 32, 347–358. 10.1016/j.molcel.2008.09.016 18995833

[B129] Rosenzweig R. Moradi S. Zarrine-Afsar A. Glover J. R. Kay L. E. (2013). Unraveling the Mechanism of Protein Disaggregation through a ClpB-DnaK Interaction. Science 339, 1080–1083. 10.1126/science.1233066 23393091

[B130] Rosenzweig R. Sekhar A. Nagesh J. Kay L. E. (2017). Promiscuous Binding by Hsp70 Results in Conformational Heterogeneity and Fuzzy Chaperone-Substrate Ensembles. eLife 6, e28030. 10.7554/elife.28030 28708484 PMC5511010

[B131] Rüdiger S. Germeroth L. Schneider-Mergener J. Bukau B. (1997). Substrate Specificity of the DnaK Chaperone Determined by Screening Cellulose-Bound Peptide Libraries. EMBO J. 16, 1501–1507. 10.1093/emboj/16.7.1501 9130695 PMC1169754

[B132] Rüdiger S. Mayer M. P. Schneider-Mergener J. Bukau B. (2000). Modulation of Substrate Specificity of the DnaK Chaperone by Alteration of a Hydrophobic Arch. J. Mol. Biol. 304, 245–251. 10.1006/jmbi.2000.4193 11090270

[B133] Rüdiger S. Schneider-Mergener J. Bukau B. (2001). Its Substrate Specificity Characterizes the DnaJ Co-chaperone as a Scanning Factor for the DnaK Chaperone. EMBO J. 20, 1042–1050. 10.1093/emboj/20.5.1042 11230128 PMC145471

[B134] Rutherford S. L. Lindquist S. (1998). Hsp90 as a Capacitor for Morphological Evolution. Nature 396, 336–342. 10.1038/24550 9845070

[B135] Sahi C. Lee T. Inada M. Pleiss J. A. Craig E. A. (2010). Cwc23, an Essential J Protein Critical for Pre-mRNA Splicing with a Dispensable J Domain. Mcb 30, 33–42. 10.1128/mcb.00842-09 19822657 PMC2798280

[B136] Sarbeng E. B. Liu Q. Tian X. Yang J. Li H. Wong J. L. (2015). A Functional DnaK Dimer Is Essential for the Efficient Interaction with Hsp40 Heat Shock Protein. J. Biol. Chem. 290, 8849–8862. 10.1074/jbc.m114.596288 25635056 PMC4423677

[B137] Sarraf N. S. Baardsnes J. Cheng J. O'Connor-McCourt M. Cygler M. Ekiel I. (2010). Structural Basis of the Regulation of the CbpA Co-chaperone by its Specific Modulator CbpM. J. Mol. Biol. 398, 111–121. 10.1016/j.jmb.2010.03.006 20226195

[B138] Sarraf N. S. Shi R. McDonald L. Baardsnes J. Zhang L. Cygler M. (2014). Structure of CbpA J-Domain Bound to the Regulatory Protein CbpM Explains its Specificity and Suggests Evolutionary Link between CbpM and Transcriptional Regulators. PLoS ONE 9, e100441. 10.1371/journal.pone.0100441 24945826 PMC4063869

[B139] Schilke B. A. Ciesielski S. J. Ziegelhoffer T. Kamiya E. Tonelli M. Lee W. (2017). Broadening the Functionality of a J-protein/Hsp70 Molecular Chaperone System. Plos Genet. 13, e1007084. 10.1371/journal.pgen.1007084 29084221 PMC5679652

[B140] Schilke B. Williams B. Knieszner H. Pukszta S. D'Silva P. Craig E. A. (2006). Evolution of Mitochondrial Chaperones Utilized in Fe-S Cluster Biogenesis. Curr. Biol. 16, 1660–1665. 10.1016/j.cub.2006.06.069 16920629

[B141] Schlecht R. Erbse A. H. Bukau B. Mayer M. P. (2011). Mechanics of Hsp70 Chaperones Enables Differential Interaction with Client Proteins. Nat. Struct. Mol. Biol. 18, 345–351. 10.1038/nsmb.2006 21278757

[B142] Schmid D. Baici A. Gehring H. Christen P. (1994). Kinetics of Molecular Chaperone Action. Science 263, 971–973. 10.1126/science.8310296 8310296

[B143] Schmid S. L. Braell W. A. Rothman J. E. (1985). ATP Catalyzes the Sequestration of Clathrin during Enzymatic Uncoating. J. Biol. Chem. 260, 10057–10062. 10.1016/s0021-9258(17)39211-6 2862148

[B144] Schönfeld H.-J. Schmidt D. Schröder H. Bukau B. (1995). The DnaK Chaperone System of *Escherichia coli*: Quaternary Structures and Interactions of the DnaK and GrpE Components. J. Biol. Chem. 270, 2183–2189. 10.1074/jbc.270.5.2183 7836448

[B145] Schramm F. D. Heinrich K. Thuring M. Bernhardt J. Jonas K. (2017). An Essential Regulatory Function of the DnaK Chaperone Dictates the Decision between Proliferation and Maintenance in *Caulobacter crescentus* . Plos Genet. 13, e1007148. 10.1371/journal.pgen.1007148 29281627 PMC5760092

[B146] Schumann W. (2016). Regulation of Bacterial Heat Shock Stimulons. Cell Stress and Chaperones 21, 959–968. 10.1007/s12192-016-0727-z 27518094 PMC5083672

[B147] Scocchi M. Tossi A. Gennaro R. (2011). Proline-rich Antimicrobial Peptides: Converging to a Non-lytic Mechanism of Action. Cel. Mol. Life Sci. 68, 2317–2330. 10.1007/s00018-011-0721-7 PMC11114787 21594684

[B148] Sekhar A. Rosenzweig R. Bouvignies G. Kay L. E. (2016). Hsp70 Biases the Folding Pathways of Client Proteins. Proc. Natl. Acad. Sci. USA 113, E2794–E2801. 10.1073/pnas.1601846113 27140645 PMC4878499

[B149] Sekhar A. Rosenzweig R. Bouvignies G. Kay L. E. (2015). Mapping the Conformation of a Client Protein through the Hsp70 Functional Cycle. Proc. Natl. Acad. Sci. USA 112, 10395–10400. 10.1073/pnas.1508504112 26240333 PMC4547247

[B150] Sekhar A. Velyvis A. Zoltsman G. Rosenzweig R. Bouvignies G. Kay L. E. (2018). Conserved Conformational Selection Mechanism of Hsp70 Chaperone-Substrate Interactions. eLife 7, e32764. 10.7554/elife.32764 29460778 PMC5819949

[B151] Sha B. Lee S. Cyr D. M. (2000). The crystal Structure of the Peptide-Binding Fragment from the Yeast Hsp40 Protein Sis1. Structure 8, 799–807. 10.1016/s0969-2126(00)00170-2 10997899

[B152] Shalgi R. Hurt J. A. Krykbaeva I. Taipale M. Lindquist S. Burge C. B. (2013). Widespread Regulation of Translation by Elongation Pausing in Heat Shock. Mol. Cel 49, 439–452. 10.1016/j.molcel.2012.11.028 PMC3570722 23290915

[B153] Sharma S. K. De Los Rios P. Christen P. Lustig A. Goloubinoff P. (2010). The Kinetic Parameters and Energy Cost of the Hsp70 Chaperone as a Polypeptide Unfoldase. Nat. Chem. Biol. 6, 914–920. 10.1038/nchembio.455 20953191

[B154] Silberg J. J. Hoff K. G. Vickery L. E. (1998). The Hsc66-Hsc20 Chaperone System inEscherichia Coli: Chaperone Activity and Interactions with the DnaK-DnaJ-GrpE System. J. Bacteriol. 180, 6617–6624. 10.1128/jb.180.24.6617-6624.1998 9852006 PMC107765

[B155] Silberg J. J. Tapley T. L. Hoff K. G. Vickery L. E. (2004). Regulation of the HscA ATPase Reaction Cycle by the Co-chaperone HscB and the Iron-Sulfur Cluster Assembly Protein IscU. J. Biol. Chem. 279, 53924–53931. 10.1074/jbc.m410117200 15485839

[B156] Silberg J. J. Vickery L. E. (2000). Kinetic Characterization of the ATPase Cycle of the Molecular Chaperone Hsc66 from *Escherichia coli* . J. Biol. Chem. 275, 7779–7786. 10.1074/jbc.275.11.7779 10713091

[B157] Singh V. K. Utaida S. Jackson L. S. Jayaswal R. K. Wilkinson B. J. Chamberlain N. R. (2007). Role for dnaK Locus in Tolerance of Multiple Stresses in *Staphylococcus aureus* . Microbiology (Reading) 153, 3162–3173. 10.1099/mic.0.2007/009506-0 17768259

[B158] Smock R. G. Blackburn M. E. Gierasch L. M. (2011). Conserved, Disordered C Terminus of DnaK Enhances Cellular Survival upon Stress and DnaK *In Vitro* Chaperone Activity*. J. Biol. Chem. 286, 31821–31829. 10.1074/jbc.m111.265835 21768118 PMC3173061

[B159] Smock R. G. Rivoire O. Russ W. P. Swain J. F. Leibler S. Ranganathan R. (2010). An Interdomain Sector Mediating Allostery in Hsp70 Molecular Chaperones. Mol. Syst. Biol. 6, 414. 10.1038/msb.2010.65 20865007 PMC2964120

[B160] Sousa R. Liao H.-S. Cuéllar J. Jin S. Valpuesta J. M. Jin A. J. (2016). Clathrin-coat Disassembly Illuminates the Mechanisms of Hsp70 Force Generation. Nat. Struct. Mol. Biol. 23, 821–829. 10.1038/nsmb.3272 27478930 PMC5016234

[B161] Sugimoto S. Arita-Morioka K. I. Terao A. Yamanaka K. Ogura T. Mizunoe Y. (2018). Multitasking of Hsp70 Chaperone in the Biogenesis of Bacterial Functional Amyloids. Commun. Biol. 1, 52. 10.1038/s42003-018-0056-0 30271935 PMC6123696

[B162] Suh W.-C. Burkholder W. F. Lu C. Z. Zhao X. Gottesman M. E. Gross C. A. (1998). Interaction of the Hsp70 Molecular Chaperone, DnaK, with its Cochaperone DnaJ. Proc. Natl. Acad. Sci. 95, 15223–15228. 10.1073/pnas.95.26.15223 9860950 PMC28024

[B163] Swain J. F. Dinler G. Sivendran R. Montgomery D. L. Stotz M. Gierasch L. M. (2007). Hsp70 Chaperone Ligands Control Domain Association via an Allosteric Mechanism Mediated by the Interdomain Linker. Mol. Cel 26, 27–39. 10.1016/j.molcel.2007.02.020 PMC1894942 17434124

[B164] Tapley T. L. Cupp-Vickery J. R. Vickery L. E. (2006). Structural Determinants of HscA Peptide-Binding Specificity†. Biochemistry 45, 8058–8066. 10.1021/bi0606187 16800630

[B165] Thompson A. D. Bernard S. M. Skiniotis G. Gestwicki J. E. (2012). Visualization and Functional Analysis of the Oligomeric States of *Escherichia coli* Heat Shock Protein 70 (Hsp70/DnaK). Cell Stress and Chaperones 17, 313–327. 10.1007/s12192-011-0307-1 22076723 PMC3312962

[B166] Tomiczek B. Delewski W. Nierzwicki L. Stolarska M. Grochowina I. Schilke B. (2020). Two-step Mechanism of J-Domain Action in Driving Hsp70 Function. Plos Comput. Biol. 16, e1007913–29. 10.1371/journal.pcbi.1007913 32479549 PMC7289447

[B167] Tsai J. Douglas M. G. (1996). A Conserved HPD Sequence of the J-Domain Is Necessary for YDJ1 Stimulation of Hsp70 ATPase Activity at a Site Distinct from Substrate Binding. J. Biol. Chem. 271, 9347–9354. 10.1074/jbc.271.16.9347 8621599

[B168] Veinger L. Diamant S. Buchner J. Goloubinoff P. (1998). The Small Heat-Shock Protein IbpB from *Escherichia coli* Stabilizes Stress-Denatured Proteins for Subsequent Refolding by a Multichaperone Network. J. Biol. Chem. 273, 11032–11037. 10.1074/jbc.273.18.11032 9556585

[B169] Vickery L. E. Cupp-Vickery J. R. (2007). Molecular Chaperones HscA/Ssq1 and HscB/Jac1 and Their Roles in Iron-Sulfur Protein Maturation. Crit. Rev. Biochem. Mol. Biol. 42, 95–111. 10.1080/10409230701322298 17453917

[B170] Vogel M. Bukau B. Mayer M. P. (2006). Allosteric Regulation of Hsp70 Chaperones by a Proline Switch. Mol. Cel 21, 359–367. 10.1016/j.molcel.2005.12.017 16455491

[B171] Vogel M. Mayer M. P. Bukau B. (2006). Allosteric Regulation of Hsp70 Chaperones Involves a Conserved Interdomain Linker. J. Biol. Chem. 281, 38705–38711. 10.1074/jbc.m609020200 17052976

[B172] Wall D. Zylicz M. Georgopoulos C. (1994). The NH2-terminal 108 Amino Acids of the *Escherichia coli* DnaJ Protein Stimulate the ATPase Activity of DnaK and Are Sufficient for Lambda Replication. J. Biol. Chem. 269, 5446–5451. 10.1016/s0021-9258(17)37706-2 8106526

[B173] Warnecke T. (2012). Loss of the DnaK-DnaJ-GrpE Chaperone System Among the Aquificales. Mol. Biol. Evol. 29(11):3485–3495. 10.1093/molbev/mss152 22683810

[B174] Waterhouse A. Bertoni M. Bienert S. Studer G. Tauriello G. Gumienny R. (2018). SWISS-MODEL: Homology Modelling of Protein Structures and Complexes. Nucleic Acids Res. 46, W296–W303. 10.1093/nar/gky427 29788355 PMC6030848

[B175] Wawrzynów A. Banecki B. Wall D. Liberek K. Georgopoulos C. Zylicz M. (1995). ATP Hydrolysis Is Required for the DnaJ-dependent Activation of DnaK Chaperone for Binding to Both Native and Denatured Protein Substrates. J. Biol. Chem. 270, 19307–19311. 10.1074/jbc.270.33.19307 7642606

[B176] Wentink A. S. Nillegoda N. B. Feufel J. Ubartaitė G. Schneider C. P. De Los Rios P. (2020). Molecular Dissection of Amyloid Disaggregation by Human HSP70. Nature 587, 483–488. 10.1038/s41586-020-2904-6 33177717

[B177] Wickner S. Hoskins J. McKenney K. (1991). Monomerization of RepA Dimers by Heat Shock Proteins Activates Binding to DNA Replication. Proc Natl Acad Sci U S A . 88(18): 7903–7907. 10.1073/pnas.88.18.7903. 1896443 10.1073/pnas.88.18.7903 PMC52413

[B178] Winter J. Linke K. Jatzek A. Jakob U. (2005). Severe Oxidative Stress Causes Inactivation of DnaK and Activation of the Redox-Regulated Chaperone Hsp33. Mol. Cel 17, 381–392. 10.1016/j.molcel.2004.12.027 15694339

[B179] Wisén S. Androsavich J. Evans C. G. Chang L. Gestwicki J. E. (2008). Chemical Modulators of Heat Shock Protein 70 (Hsp70) by Sequential, Microwave-Accelerated Reactions on Solid Phase. Bioorg. Med. Chem. Lett. 18, 60–65. 10.1016/j.bmcl.2007.11.027 18060774

[B180] Wisén S. Bertelsen E. B. Thompson A. D. Patury S. Ung P. Chang L. (2010). Binding of a Small Molecule at a Protein-Protein Interface Regulates the Chaperone Activity of Hsp70-Hsp40. ACS Chem. Biol. 5, 611–622. 10.1021/cb1000422 20481474 PMC2950966

[B181] Wisén S. Gestwicki J. E. (2008). Identification of Small Molecules that Modify the Protein Folding Activity of Heat Shock Protein 70. Anal. Biochem. 374, 371–377. 10.1016/j.ab.2007.12.009 18191466

[B182] Wu C.-C. Naveen V. Chien C.-H. Chang Y.-W. Hsiao C.-D. (2012). Crystal Structure of DnaK Protein Complexed with Nucleotide Exchange Factor GrpE in DnaK Chaperone System. J. Biol. Chem. 287, 21461–21470. 10.1074/jbc.m112.344358 22544739 PMC3375567

[B183] Wu N. He L. Cui P. Wang W. Yuan Y. Liu S. (2015). Ranking of Persister Genes in the Same *Escherichia coli* Genetic Background Demonstrates Varying Importance of Individual Persister Genes in Tolerance to Different Antibiotics. Front. Microbiol. 6, 1003. 10.3389/fmicb.2015.01003 26483762 PMC4588708

[B184] Wu Y. Li J. Jin Z. Fu Z. Sha B. (2005). The crystal Structure of the C-Terminal Fragment of Yeast Hsp40 Ydj1 Reveals Novel Dimerization Motif for Hsp40. J. Mol. Biol. 346, 1005–1011. 10.1016/j.jmb.2004.12.040 15701512

[B185] Yang J. Nune M. Zong Y. Zhou L. Liu Q. (2015). Close and Allosteric Opening of the Polypeptide-Binding Site in a Human Hsp70 Chaperone BiP. Structure 23, 2191–2203. 10.1016/j.str.2015.10.012 26655470 PMC4680848

[B186] Yang J. Yan R. Roy A. Xu D. Poisson J. Zhang Y. (2015). The I-TASSER Suite: Protein Structure and Function Prediction. Nat. Methods 12, 7–8. 10.1038/nmeth.3213 25549265 PMC4428668

[B187] Yang J. Zhang Y. (2015). I-TASSER Server: New Development for Protein Structure and Function Predictions. Nucleic Acids Res. 43, W174–W181. 10.1093/nar/gkv342 25883148 PMC4489253

[B188] Yang J. Zong Y. Su J. Li H. Zhu H. Columbus L. (2017). Conformation Transitions of the Polypeptide-Binding Pocket Support an Active Substrate Release from Hsp70s. Nat. Commun. 8, 1201. 10.1038/s41467-017-01310-z 29084938 PMC5662698

[B189] Yu H. Y. Ziegelhoffer T. Craig E. A. (2015). Functionality of Class A and Class B J-Protein Co-chaperones with Hsp70. FEBS LETTERS 589, 2825–2830. 10.1016/j.febslet.2015.07.040 26247431 PMC4570866

[B190] Zahn M. Berthold N. Kieslich B. Knappe D. Hoffmann R. Sträter N. (2013). Structural Studies on the Forward and Reverse Binding Modes of Peptides to the Chaperone DnaK. J. Mol. Biol. 425, 2463–2479. 10.1016/j.jmb.2013.03.041 23562829

[B191] Zhu X. Zhao X. Burkholder W. F. Gragerov A. Ogata C. M. Gottesman M. E. (1996). Structural Analysis of Substrate Binding by the Molecular Chaperone DnaK. Science 272, 1606–1614. 10.1126/science.272.5268.1606 8658133 PMC5629921

[B192] Zhuravleva A. Clerico E. M. Gierasch L. M. (2012). An Interdomain Energetic Tug-Of-War Creates the Allosterically Active State in Hsp70 Molecular Chaperones. Cell 151, 1296–1307. 10.1016/j.cell.2012.11.002 23217711 PMC3521165

[B193] Żwirowski S. Kłosowska A. Obuchowski I. Nillegoda N. B. Piróg A. Ziętkiewicz S. (2017). Hsp70 Displaces Small Heat Shock Proteins from Aggregates to Initiate Protein Refolding. EMBO J. 36, 783–796. 10.15252/embj.201593378 28219929 PMC5350560

[B194] Zylicz M. Ang D. Liberek K. Georgopoulos C. (1989). Initiation of Lambda DNA Replication with Purified Host- and Bacteriophage-Encoded Proteins: the Role of the dnaK, dnaJ and grpE Heat Shock Proteins. EMBO J. 8, 1601–1608. 10.1002/j.1460-2075.1989.tb03544.x 2527744 PMC400992

